# Comparing the stability and reproducibility of brain-behavior relationships found using canonical correlation analysis and partial least squares within the ABCD sample

**DOI:** 10.1162/netn_a_00363

**Published:** 2024-07-01

**Authors:** Hajer Nakua, Ju-Chi Yu, Hervé Abdi, Colin Hawco, Aristotle Voineskos, Sean Hill, Meng-Chuan Lai, Anne L. Wheeler, Anthony Randal McIntosh, Stephanie H. Ameis

**Affiliations:** Campbell Family Mental Health Research Institute, Centre for Addiction and Mental Health, Toronto, Ontario, Canada; Institute of Medical Science, University of Toronto, Toronto, Ontario, Canada; Simon Fraser University, Vancouver, British Columbia, Canada; The University of Texas at Dallas, Richardson, TX, USA; Program in Neurosciences and Mental Health, The Hospital for Sick Children, Ontario, Canada; Department of Psychiatry, Temerty Faculty of Medicine, University of Toronto, Toronto, Ontario, Canada

**Keywords:** Brain-behavior relationships, Multivariate modeling, Population-based samples, Cortical thickness

## Abstract

Canonical correlation analysis (CCA) and partial least squares correlation (PLS) detect linear associations between two data matrices by computing latent variables (LVs) having maximal correlation (CCA) or covariance (PLS). This study compared the similarity and generalizability of CCA- and PLS-derived brain-behavior relationships. Data were accessed from the baseline Adolescent Brain Cognitive Development (ABCD) dataset (*N* > 9,000, 9–11 years). The brain matrix consisted of cortical thickness estimates from the Desikan-Killiany atlas. Two phenotypic scales were examined separately as the behavioral matrix; the Child Behavioral Checklist (CBCL) subscale scores and NIH Toolbox performance scores. Resampling methods were used to assess significance and generalizability of LVs. LV_1_ for the CBCL brain relationships was found to be significant, yet not consistently stable or reproducible, across CCA and PLS models (singular value: CCA = .13, PLS = .39, *p* < .001). LV_1_ for the NIH brain relationships showed similar relationships between CCA and PLS and was found to be stable and reproducible (singular value: CCA = .21, PLS = .43, *p* < .001). The current study suggests that stability and reproducibility of brain-behavior relationships identified by CCA and PLS are influenced by the statistical characteristics of the phenotypic measure used when applied to a large population-based pediatric sample.

## Author Summary

Clinical neuroscience research is going through a translational crisis largely due to the challenges of producing meaningful and generalizable results. Two critical limitations within clinical neuroscience research are the use of univariate statistics and between-study methodological variation. Univariate statistics may not be sensitive enough to detect complex relationships between several variables, and methodological variation poses challenges to the generalizability of the results. We compared two widely used multivariate statistical approaches, canonical correlations analysis (CCA) and partial least squares correlation (PLS), to determine the generalizability and stability of their solutions. We show that the properties of the measures inputted into the analysis likely play a more substantial role in the generalizability and stability of results compared to the specific approach applied (i.e., CCA or PLS).

## INTRODUCTION

Altered brain structure has been linked to elevated psychopathology symptoms across clinical and nonclinical samples (Albaugh et al., 2017; Ameis et al., 2014; Ducharme et al., 2014; Jacobs et al., 2021; Mihalik et al., 2019; Qin et al., 2014; Romer et al., 2021). Yet, brain-behavior associations have shown poor replicability across studies (Boekel et al., 2015; Lombardo et al., 2019; Masouleh et al., 2019), with recent work suggesting thousands of participants are required for replicable findings in cross-sectional studies (Marek et al., 2019). Use of small sample sizes, between-study methodological variation, and underpowered statistical approaches have contributed to the lack of consistent results between studies (Button et al., 2013; Grady et al., 2021; Lombardo et al., 2019; Poldrack et al., 2017). To address the limitations associated with small sample sizes, recent multisite initiatives such as the Adolescent Brain Cognitive Development (ABCD) study have provided the capacity to use thousands of participants with rich multidimensional data. However, between-study methodological variation makes it difficult to draw conclusions about the generalizability of brain-behavior relationships. Studies that systematically compare different statistical approaches can help guide decisions around which approaches are most suitable for identifying stable and reproducible brain-behavior associations. Recent research has noted several advantages of multivariate approaches over univariate approaches (Marek et al., 2022; Masouleh et al., 2019) likely due to the limited power and sensitivity of univariate methods when applied to complex brain-behavior data (Mardia et al., 1979; McIntosh, 2021; Nakua et al., 2022).

Two multivariate approaches that are widely used to examine the association between two sets of variables are canonical correlations analysis (CCA) and partial least squares correlation (referred to as PLS, not be confused with PLS-regression; Krishnan et al., 2011). These approaches derive a set of latent variable (LV) pairs, in which each pair is composed of a linear combination of variables from one data matrix, so that LVs have maximizing correlation (CCA) or covariance (PLS) (Hotelling, 1936; Wold, 1985). Both CCA and PLS have been widely applied to clinical and population-based samples to analyze brain metrics and phenotypic measures (Avants et al., 2014; Drysdale et al. 2017; Ing et al., 2019; Itahashi et al., 2020; Kebets et al., 2019; Mihalik et al., 2019; Modabbernia et al., 2021; Seok et al., 2021; Smith et al., 2015; Wang et al., 2018; Xia et al., 2018; Ziegler et al., 2013). However, studies do not typically provide justification for choosing between these methods, making it unclear whether one approach is better suited for particular analytic contexts (e.g., when using specific phenotypic scales).

Two prior studies have systematically compared the results from CCA and PLS to determine the strengths, weaknesses, and similarities of both approaches (Helmer et al., 2024; McIntosh, 2021). Helmer et al. (2024) found that both CCA and PLS findings require an adequate sample size (*N* > 1,000) to be stable and reliable. McIntosh (2021) found that relationships are most similar between CCA and PLS when the correlations within each data matrix are low. Although these studies provide insight into the different use-cases and outputs of CCA and PLS, an unaddressed gap is determining whether characteristics of a dataset (e.g., measurements used, sample characteristics) influence the reliability of the models derived from CCA or from PLS, particularly in widely used open-access datasets.

To address this gap, we employed a data-driven analytical design that applied CCA and PLS to brain and phenotypic behavior data in the ABCD dataset to (1) examine the similarity between the identified LVs derived from CCA and PLS when delineating brain-behavior relationships (between-method generalizability), and (2) compare the stability, reproducibility, and reliability of the LVs derived from CCA and PLS (within-method generalizability). We analyzed two widely used phenotypic measures: the Child Behavior Checklist (CBCL)—a parent-report measure of childhood behaviors relevant to various mental health diagnoses—and the NIH Cognitive Toolbox—a performance-based measure capturing various dimensions of cognition. Although the CBCL and the NIH Cognitive Toolbox have each been widely used to delineate brain-behavior relationships across different datasets (Albaugh et al., 2017; Ameis et al., 2014; Burgaleta et al., 2014; Ducharme et al., 2014; Ehrlich et al., 2012; Ronan et al., 2020), there is limited systematic comparison between the two phenotypic scales. Such a comparison will provide insight as to whether one scale may be optimal to use in multivariate brain-behavior relationship research to improve the generalizability of findings. Furthermore, using these two phenotypic measures, we are able to compare whether the generalizability of CCA and PLS is influenced by the choice of measurement (i.e., parent report or performance-based) and construct of interest (i.e., psychopathology or cognitive performance).

## METHODS

### Sample

The ABCD dataset is a longitudinal multisite population-based sample collecting a comprehensive measurement battery (including genetic, blood, environmental, cognitive, brain, and behavioral measures) in over 11,000 participants ages 9–11 years old. Data collection time points occurring annually or biannually for 10 years. Participants were recruited from 21 academic sites across the United States using probability sampling to ensure that demographic trends across the United States are well represented in the sample (see Casey et al., 2018, for more details). Recruitment occurred through presentations and emails delivered to parents of children in local schools around each site. Interested parents underwent a telephone screening to determine whether their children were eligible to participate in the study. Participants were excluded from participation in the ABCD study if they had magnetic resonance imaging contraindications, no English fluency, uncorrected vision or hearing impairments, major neurological disorders, were born extremely preterm (less than 28 weeks gestation), low birth weight (< 1,200 grams), birth complications, or unwillingness to complete assessments. The current study used tabulated data from the baseline sample provided by the ABCD consortium from the fourth annual release (DOI:10.15154/1523041). The current study excluded participants with poor brain imaging quality, missing T1-weighted scans, missing behavioral data, and all but one sibling per family if the family enrolled multiple siblings, twins, or triplets (see Supporting Information Figure S1 for consort diagram and details of exclusion). As a result, the current study analyzed 9,191 participants in the CBCL brain analysis (ages 9–11 years, 4,369 assigned female at birth, and 4,822 assigned male at birth) and 9,034 participants in the NIH brain analysis (4,292 assigned female at birth and 4,742 assigned male at birth).

### Scanning Acquisition and Processing

The neuroimaging protocol and specific T1-weighted parameters are detailed in previous publications (Casey et al., 2018; Hagler et al., 2019). Briefly, the ABCD protocol is harmonized for Siemens, General Electric, and Philips 3 T scanners. All scanners used multichannel coils capable of multiband echo planar imaging (EPI) acquisitions. The scanning occurred in either one or two sessions depending on the scanning site. Participants underwent a mock scanning session before the actual scan to help them get accustomed to the scanning environment. T1-weighted scans were collected, processed, and analyzed by the Data Analysis, Informatics Resource Center (DAIRC) based on standardized ABCD protocols (see details: Hagler et al., 2019). Cortical and subcortical segmentation was performed using FreeSurfer v5.3.0 (Dale et al., 1999; Fischl & Dale, 2000). All T1-weighted scans were examined by trained visual raters who rated each scan from 0 to 3 based on motion, intensity homogeneity, white matter underestimation, pial overestimation, and visual artifacts. From these ratings, participants with poor quality scans were recommended for exclusion by the DAIRC and were excluded from this study (for more details, see Hagler et al., 2019).

### Brain Measures

Cortical thickness derived from 68 cortical regions from the Desikan-Killiany Atlas parcellations (Desikan et al., 2006) were used as the structural morphology measures in the current study. Cortical thickness has been extensively linked to various behavioral and clinical measures in pediatric samples (e.g., Albaugh et al., 2017; Ameis et al., 2014; Ducharme et al., 2014; Vijayakumar et al., 2017), including in the ABCD sample (Laurent et al., 2020; Modabbernia et al., 2021; Yu et al., 2021).

### Behavioral Measures

Two different behavioral measures were selected for this analysis: CBCL subscale scores and the NIH Cognitive Toolbox. Both measures have been linked to structural brain morphology (e.g., Albaugh et al., 2017; Ameis et al., 2014; Burgaleta et al., 2014; Ducharme et al., 2014; Ehrlich et al., 2012; Ronan et al., 2020) and widely studied in the ABCD dataset (e.g., Gong et al., 2021; Ho et al., 2022; Huber et al., 2022; Roffman et al., 2021; Pagliaccio et al., 2021). The CBCL provides standardized parent-reported measures of behavioral symptoms relevant to mental health diagnoses (e.g., internalizing symptoms relevant to anxiety and depressive disorders), while the NIH Cognitive Toolbox offers a set of standardized performance-based measures of cognitive phenotypes (e.g., a working memory task).

In the CBCL brain analysis, we used the unadjusted scores from the CBCL as the behavioral variable set. The CBCL is a well-validated tool to assess mental health symptoms in children from a parent/caregiver report (Achenbach & Ruffle, 2000). The CBCL consists of 113 questions about a child’s behavior on an ordinal scale (0 = never, 1 = sometimes, 2 = often). The ordinal ratings are summed to provide subscale scores for a variety of behaviors (e.g., aggression). We used the eight conventional subscale scores from the parent-report CBCL for 6–18 year olds: anxious/depressed, withdrawn/depressed, somatic complaints, thought problems, social problems, rule-breaking behavior, aggressive behavior, attention problems, in addition to three subscales included in ABCD that address symptoms closely related to neurodevelopmental disorders: stress symptoms, obsessive compulsive problems, and sluggish-cognitive-tempo (Jacobson et al., 2012; Storch et al., 2006).

In the NIH brain analysis, we used the unadjusted scores from the NIH Cognitive Toolbox as the behavioral variable set. The NIH Toolbox consists of seven tasks presented on an iPad and measures five broad cognitive domains: picture vocabulary task (language skills), oral reading recognition task (language skills), list sorting working memory task (working memory), picture sequence memory task (episodic memory), flanker task (attention/inhibition), dimensional card change sort task (cognitive flexibility), and pattern comparison processing speed task (visual processing) (Thompson et al., 2019; Weintraub et al., 2013).

### Statistical Analysis

To remove the variance associated with age, sex assigned at birth, total head size, site (*N* = 21), and MRI scanner model (*N* = 5) from the results, we regressed out (i.e., partialled out) these variables from both the brain and behavioral matrix using a linear regression. The resulting residuals were *Z*-transformed (mean-centered and standard deviation of 1) and used in the CCA and PLS analyses.

#### Performing the CCA and PLS analyses.

**Figure 1.**
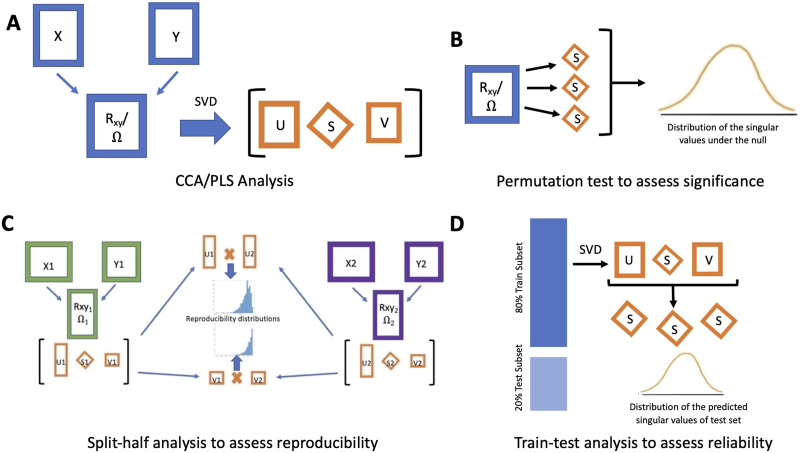
Overview of the analytical pipeline used in the current study. (A) The main CCA and PLS analysis: the decomposition of the cross-product matrices to produce singular values and loadings (reweighted singular vectors in the case of CCA). (B) The sum of squares permutation analysis to assess the statistical significance of each LV. (C) The split-half analysis used to assess the similarity between respective loadings from each split-half. In this analysis, the brain and behavioral data are both split into two different matrices (resulting in X_1_, X_2_, Y_1_, Y_2_) for each of the 10,000 iterations. Each respective split pair (e.g., X_1_ and Y_1_) then undergoes the CCA or PLS analysis shown in panel A. The resulting loadings from the respective split pairs are correlated (e.g., X_1_ and X_2_) to provide a distribution of Pearson correlation coefficients between each respective brain and behavior loading across the 10,000 iterations. (D) The train-test resampling analysis that assesses how well the singular values from the training sample can predict the singular values of the test sample. For each of the 10,000 iterations, the dataset was split into an 80% train set (X_80_, Y_80_) and 20% test set (X_20_, Y_20_). The train set underwent the CCA or PLS analysis shown in panel A. The respective loadings from the SVD of the training set were used to solve for the singular values (S) of the test set. The resulting predicted singular values of each of the 10,000 train/test analyses were plotted as a distribution. Importantly, each analysis is independent from one another (i.e., the results of one analysis are not used in another) and not sequential (i.e., the permutation test in panel B did not necessarily need to happen prior to the split-half or train-test analysis). The bootstrap confidence interval estimation, not shown here, was used to assess the stability of the parameter estimates of variable weights in the loadings. SVD = singular value decomposition.

CCA and PLS are unsupervised learning algorithms that identify maximum linear relationships between two variable sets (here a brain matrix, **X**, and a behavior matrix, **Y**; Figure 1A). These methods differ in the shared information they maximize between **X** and **Y** by decomposing a cross-product matrix under different constraints. Usually, in CCA and PLS, the variables (i.e., columns) in **X** and **Y** are mean centered with unit variance (standard deviation of 1) or have a Euclidean norm of 1 (i.e., the sum of squares equal to 1). As a result of this scaling, the cross-product matrix decomposed by PLS is proportional to the Pearson correlation matrix between **X** and **Y** (denoted by **R**_**XY**_). In contrast, the cross-product matrix decomposed by CCA is the *adjusted* Pearson correlation matrix between **X** and **Y** (denoted by **Ω**) as it is normalized by the within-block correlations of **X** and of **Y**. This difference in adjustment of within-block correlation makes the relationships derived from CCA optimized for correlation and those derived from PLS optimized for covariance (see details in Supporting Information Section 1, and in prior work: Abdi et al., 2017; Krishnan et al., 2011; McIntosh, 2021).

CCA and PLS maximize the associations between two data matrices by decomposing the **R**_**XY**_ or **Ω** matrix using the singular value decomposition (SVD) algorithm. The SVD decomposes **R**_**XY**_ or **Ω** into two matrices of orthonormal singular vectors (**U** and **V**; in the CCA literature, these are denoted as generalized singular vectors, see Supporting Information Section 1) and a diagonal singular value matrix (**S**). The **U** and **V** singular vectors store the **X** and the **Y** *loadings* that characterize the association between the two data matrices, respectively. The relationship between the respective **U** and **V** singular vectors (henceforth will be denoted as loadings) comprises pairs of *latent variables* (LVs; analogous to latent dimensions in the multivariate literature; Krishnan et al., 2011). The loadings represent the weight of each variable in each LV (i.e., the degree to which that variable contributes to the latent relationship). For PLS, the loadings are directly derived from the **U** and **V** matrices, whereas for CCA, they are reweighted (see Supporting Information Section 1 for details). To better understand the latent relationships between **X** and **Y** at the participant level, each matrix is projected onto the respective loadings to create *latent scores* (i.e., **XU** and **YV**; analogous to factor scores). For each LV, the **XU-YV** pair of scores are optimized for correlation (in CCA) or covariance (in PLS) to describe the multivariate covariation between the **X** and the **Y** matrices. The singular values (from the **S** matrix) quantify the strength of the relationships captured by the corresponding pairs of latent scores from **XU** and **YV**. The largest possible number of LVs generated is equivalent to the minimum number of variables between the two matrices (e.g., 11 LVs in the CBCL brain analyses).

The structure and distribution of **X** or **Y** may require some preprocessing prior to calculating the cross-product matrix. In the current sample, the CBCL data were skewed with a high proportion of the sample having zero or low scores across subscales. As a result, to remain consistent between the CBCL brain and NIH brain analysis, cross-product matrices (**R**_**XY**_ and **Ω**) were implemented using Spearman’s correlation given that the nonparametric Spearman’s correlation coefficient is robust against skewed data (de Winter et al., 2016; Myers & Sirois, 2004).

To assess the significance of the LVs identified from CCA and PLS, we performed a permutation test on the singular values (McIntosh & Lobaugh, 2004; McIntosh, 2021). Briefly, the brain matrix underwent 10,000 iterations of resampling without replacement. Then, each resampled brain matrix and original behavioral matrix underwent the CCA or PLS analytical pipeline (Figure 1B). The permutation resampling procedure breaks the associations between the brain and behavior matrices creating a cross-product matrix under a null distribution. Given the large sample size, a standard permutation test may not be stringent enough to detect significantly meaningful LVs. To impose stringency on the detection of significant LVs, we performed a “sum of squares” permutation test (analogous to Wilk’s lambda). This test assesses the *eigenspectrum* (i.e., a set of eigenvalues that are the square of the singular values) of CCA or PLS models to determine if the total variance explained by a given set of LVs occurs greater than expected by chance. It works by generating the sum of squares for *K* − 1 singular values (with 1 ≤ *K* < 11 [i.e., LV_1_–LV_11_, then LV_2_–LV_11_, etc.]). This permutation test generates null distributions of a given eigenspectrum depicting the likelihood of brain-behavior relationships captured by a given LV occurring at random. Each LV was significant if less than 5% of the permuted sum of square singular values (e.g., LV_1_–LV_11_ for LV_1_, LV_2_–LV_11_ for LV_2_, etc.) were equal or greater than the empirical sum of squared singular values. Between-method generalizability was quantitatively assessed using a Pearson correlation between the respective **U** and **V** loadings as derived from CCA and PLS models.

#### Assessing reproducibility, reliability, and stability.

Resampling statistics were used to assess reproducibility, reliability, and stability of the LVs identified. We defined reproducibility as ‘obtaining similar results in a different sample’ which was assessed using split-half resampling of the loadings (Figure 1C). We defined reliability as ‘ability to accurately predict results on unseen data’ assessed using train-test resampling of the singular values (Figure 1D). We defined stability as ‘results that can best approximate the relationship in the population,’ which was assessed using bootstrap resampling of elements (i.e., individual variables). The split-half and train-test resampling analyses were conducted using 10,000 iterations. The bootstrap resampling was conducted using 1,000 iterations due to computational constraints. Reproducible and reliable distributions were identified using a *Z*-test (i.e., mean divided by standard deviation of distribution). A *Z*-test magnitude > 1.96 is associated with a *p* value < .05, a pattern indicating that the distribution significantly differed from zero. Prior work using simulated data confirms that randomly permuted distributions rarely exceed a *Z*-score of 1.65 (which would match a *p* value of .10), a configuration suggesting that this cutoff is appropriate to use to determine significantly reproducible distributions (McIntosh, 2021). For all resampling analyses, the linear regression analysis to remove the effects of site, age, sex assigned at birth, total head size, and scanner manufacturer was performed following the resampling or splitting of participants.

#### Assessing reproducibility with split-half resampling.

For each iteration, the original **X** and **Y** matrices were randomly split into two halves to create four matrices (i.e., **X**_**1**_ and **Y**_1_ for the first half, and **X**_**2**_ and **Y**_**2**_ for the second half) conserving the relationship between **X** and **Y** for each resample (Churchill et al., 2013). CCA and PLS analysis were separately performed in each half (e.g., within a single iteration, CCA was conducted between **X**_**1**_ and **Y**_**1**_, and then separately between **X**_**2**_ and **Y**_**2**_). A Pearson correlation was used to assess the relationship between **U** and **V** loadings from the halves of each iteration (e.g., **U**_**1**_ and **U**_**2**_ from the analysis of the first and the second halves). Higher correlations are indicative of similar respective loadings from each split-half within a single iteration. The *Z*-test was performed on the distribution of Pearson correlation values between each of the 11 (or 7 in the case of the NIH brain analysis) loadings of matrices **U** and **V**. A significant Pearson correlation coefficient distribution would suggest that regardless of the participants included in the split-half, the results of the CCA- and/or PLS-derived latent brain-behavior relationship would be consistent. This pattern, then, suggests that the loadings of the main analyses are not influenced by characteristics of a subset of participants.

#### Assessing reliability with train-test resampling.

For each iteration, the data is split at random into an 80/20 train/test split. The CCA and PLS analyses were initially performed on the training set, and the loadings were projected onto the cross-product matrix of the test set to solve for the singular values (details in McIntosh, 2021). This test assesses the magnitude of the association between **X** and **Y**. A predicted singular value distribution that is significantly and consistently larger than 0 indicates that the derived outputs from the 80% training set can reliably predict the mean of the singular values of the 20% test set. The *Z*-test was performed on the distribution of the predicted singular values.

#### Assessing stability with bootstrap resampling.

Stability of the elements (i.e., individual variables, e.g., anxiety/depression subscale score from the CBCL) within a single LV was assessed using bootstrap resampling of the loadings. Using a Monte-Carlo bootstrap approach, the data matrices **X** and **Y** were generated 1,000 times by randomly selecting the participants with replacement until the total sample size was reached (*N* = 9,191 or 9,034) while conserving the relationship between **X** and **Y**. The CCA and PLS were separately conducted for each regenerated **X** and **Y** matrix, a procedure resulting in 1,000 regenerated matrices of loadings **U** and **V**. We corrected for arbitrary sign flips that often occur in iterations of the SVD (see Supporting Information Section 1.2 for details). The 1,000 regenerated **U** and **V** loadings provide *bootstrap samples* that were used to create the *bootstrapped distribution* of each element in **U** and **V**. From these bootstrapped distributions, we computed the 95% *bootstrapped* confidence intervals of each element in **U** and **V** to quantify their stability. A stable variable in each LV was determined by a corresponding 95% bootstrapped confidence interval that does not include zero.

### Sensitivity Analyses

We ran two sensitivity analyses to determine whether significance and reproducibility would be impacted by socioeconomic status (SES) or history of head injury given prior work suggesting these variables influence brain and behavioral metrics (Lawson et al., 2013; Piccolo et al., 2016; Wilde et al., 2012). For both these samples, we performed the CCA and PLS analysis, permutation test, and split-half resampling analysis. We analyzed a subset of the sample with available household income data (*N* = 8,399), which we used as a proxy for SES (Hill et al., 2016; Rakesh et al., 2021). In this analysis, household income data were included as a covariate in the linear regression model to extract residuals from the brain and behavior matrices. In a separate analysis, we performed CCA and PLS in a subset of the sample including participants with no history of head injuries (*N* = 8,139; see details in Supporting Information Section 3).

### Post Hoc Analyses

Given the skew of the CBCL data, within-method generalizability may be improved by stratifying the sample based on clinical severity when examining the relationship between cortical thickness and CBCL scores. We therefore implemented three post hoc analyses to explore whether variations of the sample or measures improves the reproducibility of brain-behavior relationships derived from CCA or PLS. First, we sought to determine whether participants with elevated psychopathology symptoms would show brain-behavior relationship patterns that may be washed out when conducting the analysis in the full sample. We stratified the sample by participants who had a total CBCL T-score (normalized for sex assigned at birth and age) > 60—a criterion proposed as a subclinical cutoff (*N* = 1,016; Biederman et al., 2020). Second, we removed participants with no endorsement (i.e., a score of 0) on any CBCL subscale to attain a sample with full subscale symptom endorsement to eliminate the zero inflation of the CBCL matrix (Byrd et al., 2021; *N* = 5,196; see Supporting Information Section 4). We performed the CCA and PLS analysis, permutation test, and split-half resampling analysis in both these subsets. These analyses provide insight as to whether using a subset of the sample with elevated clinical scores may be more reliable in elucidating meaningful and generalizable brain-behavior relationships when using clinical scales. Third, we compared CCA- and PLS-derived brain-behavior relationships when using additional clinical and cognitive measures to probe whether these methods would be largely impacted by the scale of measurement. For the clinical behavioral matrix, we included subscale scores from the self-report scale, the Behavioral Inhibition/Behavioral Approach System (BIS/BAS; Pagliaccio et al., 2016) and the parent-report UPPS-P for Children’s Survey (Lynam et al., 2007; Watts et al., 2020). For the cognitive behavioral matrix, we included performance scores from the Rey Auditory Verbal Learning Test (Strauss et al., 2006). We performed several analyses to compare correlation structure, distribution skew, cross-product matrices, and reproducibility of loadings (Supporting Information Section 3.6).

### Data and Code Availability

Data for the ABCD Study are available through the National Institutes of Health Data Archive (NDA; nih.nda.gov). The participant IDs included in these analyses and details on the measures used, can be found in this project’s NDA study (DOI:10.15154/1528644). The code for the analysis can be found on GitHub (https://github.com/hajernakua/cca_pls_comparison_ABCD). All analyses were conducted on Rstudio (v4.0.3).

## RESULTS

### Participant Characteristics

Table 1 provides the demographic details of the ABCD sample used in the current study. There were no substantial differences between the sex, household income, race/ethnicity, parental education, and behavior measures between the analyzed sample (*N* = 9,191) and the total sample acquired from ABCD with complete data (*N* = 11,804). See Supporting Information Table S1 comparing diagnostic characteristics between the sample with full CBCL data (*N* = 9,191) and analyzed subsamples (*N* = 8,399, 8,139) included in sensitivity analyses (Supporting Information Section 3 for details on sensitivity analyses). See Table S2 comparing diagnostic characteristics between participants who were included (*N* = 9,191) versus excluded (*N* = 2,613) from the current study.

**Table 1.** Demographic characteristics of the ABCD subsample included in the current study and the acquired ABCD sample

	**Included ABCD sample (*N* = 9,191)**		**Acquired ABCD sample (*N* = 11,804)**	
	**Mean [range]**	***SD***	**Mean [range]**	***SD***
Age (in months)	118.9 [107–133]	7.4	118.9 [107–133]	7.5
CBCL total T-score	46.12 [24–83]	11.4	45.84 [24–83]	11.3
CBCL internalizing T-score	48.73 [33–93]	10.7	48.45 [33–93]	10.64
CBCL externalizing T-score	45.87 [33–84]	10.4	45.73 [33–84]	10.32
	**Total**	**%**	**Total**	**%**
Sex (female)	4,369	47.5	5,637	47.7
*Household income*				
< $50K	2,531	27.5	3,196	27.1
$50–$100K	2,381	25.9	3,057	25.9
> $100K	3,487	37.9	4,544	38.5
*Participant race/ethnicity*				
White	4,704	51.2	6,150	52.1
Black	1,360	14.8	1,755	14.9
Asian	205	2.2	251	2.12
Hispanic	1,973	21.5	2,401	20.3
Other	948	10.3	1,244	10.5
*Parent education*				
< HS diploma	468	5.1	583	4.9
HS diploma	887	9.65	1,120	9.5
Some college	2,389	25.9	3,056	25.9
Bachelors	2,287	24.9	3,003	25.4
Postgraduate	3,150	34.3	4,027	34.1

*Note*. Some participants had missing household income, race/ethnicity, or parent education information, thus, the total number of participants in each category reflect those with available data. *SD* = standard deviation; CBCL = Child Behavior Checklist, HS = high school. Internalizing behavior is the summed broadband measure that includes anxious/depressed, withdrawn/depressed, and somatic complaints scores. Externalizing behavior is the summed broadband measure that includes aggressive and rule-breaking behavior. These broadband measures are clinically meaningful; however, they do not allow the exploration of brain-behavior relationships at the symptom level. The NIH brain analysis included all the participants in the CBCL brain analysis with available NIH Cognitive Toolbox data (*N* = 9,034). There were limited differences in demographic characteristics between the sample with complete CBCL data and the sample with complete NIH data.

### CCA and PLS Analyses

#### CBCL brain analysis.

**Figure 2.**
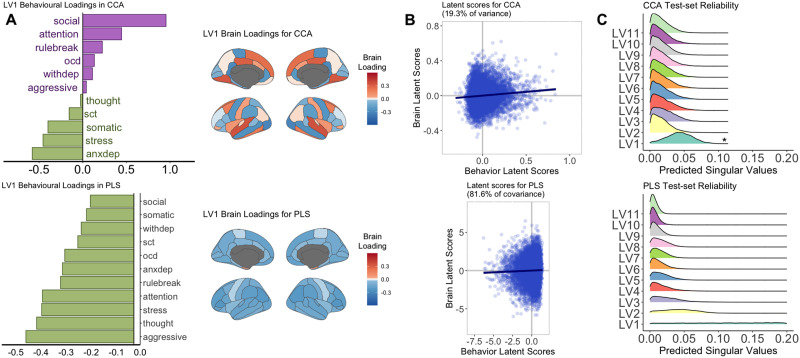
Unthresholded behavior and brain loadings from the PLS and CCA analysis performed in the CBCL brain analysis (*N* = 9,191). The highest PLS-derived behavior loadings were aggressive behavior, thought problems, and stress problems. The highest PLS-derived brain loadings were right pars triangularis, right inferior parietal cortex, and left posterior cingulate cortex. The highest CCA-derived behavior loadings were social problems, anxious/depressive symptoms, and stress problems. The highest CCA-derived brain loadings were the right superior temporal gyrus, left fusiform gyrus, and right lingual gyrus. Panel B shows the latent scores between *XU* and *YV* for LV_1_. Prior to calculating the latent scores, the brain and behavioral loadings have been standardized by the singular values. Panel C depicts the train-test distributions of the predicted singular values of the test sample for each iteration. Asterisks indicate the LVs that showed a distribution with a *Z*-score greater than 1.96. LV_1_ from CCA was found to be reliable (i.e., LV_1_ of the training sample can reliably predict the singular values of LV_1_ from the test sample). The lack of any other significant distributions of predicted singular values suggest that the 80% train set does not reliably and consistently predict the singular values from the 20% test set. OCD = obsessive compulsive symptoms; withdep = withdrawn/depression symptoms; sct = sluggish-cognitive-tempo; anxdep = anxious/depressive symptoms; rulebreak = rule-breaking behavior.

The sum of squares permutation test revealed one significant LV for both CCA and PLS (*p* < .001; Table S3) when decomposing the Spearman’s cross-product correlation matrix. The relationship between the brain (**U**) and behavior loadings (**V**) in LV_1_ was stronger in PLS compared to CCA (singular values: PLS = .39 [81.6% of covariance], CCA = .13 [19.3% of variance]). Figure 2 depicts unthresholded behavior and brain loadings for PLS and CCA. LV_1_ from PLS identified an association between lower CBCL scores (i.e., less behavioral problems) and lower cortical thickness; the strongest relationship was between aggressive behaviors and the right pars triangularis. LV_1_ from CCA identified covariation in both the brain and behavior loadings; the strongest relationship was between social problems and thickness of the right superior temporal gyrus (Figure 2A). Importantly, the interpretation of the loading direction is relative. As such, the PLS results can be interpreted as either negative behavioral loadings that are linked to negative brain loadings or as positive behavioral loadings that are linked to positive brain loadings, as long as the relationship of the sign between the brain and behavioral loadings remains the same. The Pearson correlation between the respective **U** and **V** CCA and PLS loadings showed a weak correlation in LV_1_ between CCA and PLS. However, there was a stronger correlation in LV_2_ (see Figure S2). See Figure S3 for CCA and PLS results when decomposing the Pearson correlation matrix. See Supporting Information Section 1.2 and Figure S4 for CCA results when implementing the structure coefficients. See Supporting Information Section 3 and Figure S5 indicating similar results found for sensitivity analyses controlling for household income and head injuries in the sample.

#### NIH brain analysis.

**Figure 3.**
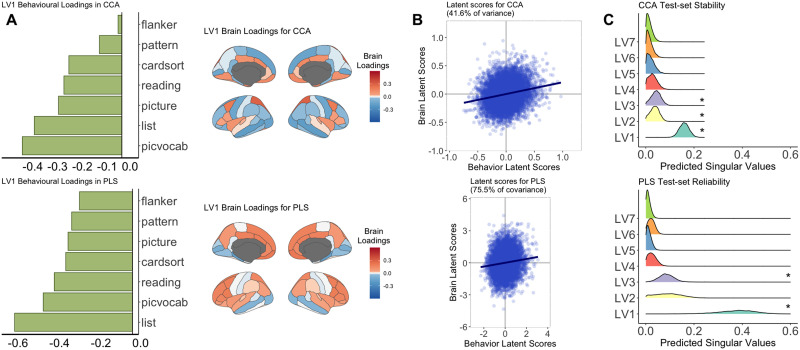
Unthresholded behavior and brain loadings of LV_1_ from the PLS and CCA analysis performed between NIH Cognitive Toolbox scores and cortical thickness. The largest PLS- and CCA-derived loadings were found for the list sorting task and the picture vocabulary task. The highest CCA-derived brain loadings were the left pars opercularis, superior frontal gyrus, and parahippocampal gyrus. The highest PLS-derived brain loadings were the left pars opercularis, parahippocampal gyrus, and medial orbitofrontal gyrus. Panel B shows the latent scores between XU and YV for LV_1_. Prior to calculating the latent scores, the brain and behavioral loadings have been standardized by the singular values. Overall, there is a similar relationship between the brain and behavioral latent scores when comparing CCA and PLS. Panel C shows the results of the train-test resampling analysis. Asterisks indicate the LVs that showed a distribution with a *Z*-score greater than 1.96. LV_1_ (*Z*-score: PLS = 4.8, CCA = 7.8) and LV_3_ (*Z*-score: PLS = 2.6, CCA = 2.2) for both PLS and CCA, and LV_2_ (*Z*-score: CCA = 2.02) for CCA, were found to be reliable (i.e., singular values of these LVs of the training sample can reliably predict the singular values from the test sample). Flanker = Flanker task; pattern = pattern comparison processing speed task; cardsort = dimensional change card sort task; reading = oral reading recognition task; picture = picture vocabulary task; list = list sorting working memory task; picvocab = picture vocabulary task.

The permutation test revealed six significant LVs (LV_1_: PLS = .43 [75.5% of variance]; CCA = .21 [41.6% of variance]; Figure 3, Table S4). In contrast to the CBCL brain analysis, LV_1_ between cortical thickness and performance on the NIH cognitive toolbox were similar between CCA and PLS—suggestive of between-method generalizability. In both CCA and PLS, this LV identified an association between decreased cognitive performance (across all variables) and higher cortical thickness in several frontal and temporal regions and lower cortical thickness in several occipital regions (Figure 3A). The strongest associations in LV_1_ were between the list sorting working memory task (working memory) and picture vocabulary task (language abilities) and the left pars opercularis and left parahippocampal gyrus. The results of the Pearson correlation between the respective **U** and **V** loadings from CCA and PLS showed moderate-to-strong positive correlations in LV_1,6_ and strong negative correlations in LV_5,7_ between CCA and PLS (see Figure S6). Nonrespective LVs between CCA and PLS showed strong correlations (e.g., loadings between LV_4_ in CCA and LV_1_ in PLS). See Supporting Information Section 3.1 and 3.2 and Figure S7 indicating similar results found for sensitivity analyses controlling for household income and head injuries in the sample.

### Reproducibility, Reliability, and Stability

#### Split-half resampling (reproducibility of loadings).

**Figure 4.**
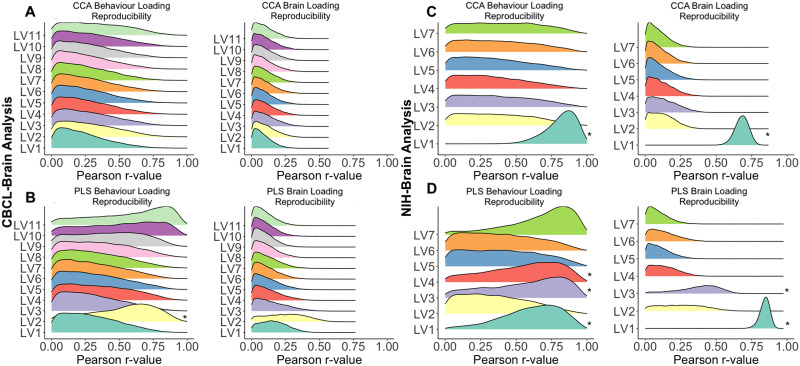
This figure depicts the distributions of the resampled loadings from the split-half analysis for the CBCL brain and NIH brain analyses. The *x*-axis from the split-half distributions are the Pearson correlation coefficients between respective loadings from each split-half analysis (e.g., *U*_1_ and *U*_2_ from the analysis comparing X_1_ and Y_1_ and separately, X_2_ and Y_2_). In the CBCL brain analysis, the distribution of Pearson correlation coefficients centered around 0 for the majority of LVs, indicating minimal correspondence between respective loadings from the split-halves. This suggests that characteristics of participants are highly influential in the loadings derived from CCA or PLS models in the CBCL brain analysis. In the NIH brain analysis, the distribution of Pearson correlation coefficients are centered around *r* = 0.6–0.8, indicating high correspondence between respective split-halves and that loadings from CCA and PLS models remain similar regardless of which participants are included in each iteration. Asterisks indicate the LVs which showed a distribution with a *Z*-score greater than 1.96.

Figure 4 shows the distributions of Pearson correlation coefficients between the **U** (or **V**) loadings from the two halves for each iteration. In the CBCL brain analysis, the *Z*-test of the Pearson correlation suggests no reproducible loadings for CCA (Figure 4B). A similar pattern was found in PLS except for the behavior loadings for LV_2_, of which the mean coefficient of correlation is significantly different from 0 (*Z*-score = 2.17), suggesting some reproducibility (Figure 4A). In the NIH brain analysis, LV_1_ for the behavioral and brain loadings was found to be reproducible for both CCA and PLS (*Z*-score range = 2.9–25). LV_3,4_ behavioral and brain loadings were found to be reproducible for PLS (*Z*-scores = 2.15, 2.57, respectively; Figure 4C and D).

#### Train-test resampling (reliability of singular values).

Figures 2C and 3C illustrate the distributions of predicted singular values of each test set iteration. In the CBCL brain analysis, the results showed that LV_1_ from CCA features a distribution significantly different from zero (*Z*-score = 2.31), indicating a reliable LV (i.e., the SVD results from the training set can reliably predict the singular values of the test set). No other LV showed reliable singular values in CCA or PLS. For the NIH brain analysis, LV_1_ and LV_3_ were found to be reliable for both CCA and PLS (*Z*-score range = 2.15–7.77), and LV_2_ was additionally found to be reliable for CCA (*Z*-score = 2.02). That is, the CCA- and PLS-derived relationships found in 80% of the sample were able to reliably predict the singular values of the remaining 20%.

#### Bootstrap resampling (stability of elements in LV_1_).

Bootstrap analysis on the loadings from the CBCL brain and NIH brain analyses revealed several stable brain and behavioral elements in LV_1_ (i.e., the 95% confidence intervals did not include zero; see Figures S8 and S9).

### Post Hoc Analyses

In the subset of the sample with elevated total CBCL scores (*T*-score > 60; *N* = 1,016), the singular values for LV_1_ were larger than the main CBCL brain analysis, a difference indicating a stronger relationship between the brain and behavioral variables (singular values of LV_1_: PLS = .67 [54% of covariance]; CCA = .33 [14.6% of variance]; see Figure S10 and Table S4). The correlations in the cross-product matrices **R**_**XY**_ and **Ω** were overall stronger than those of the main CBCL brain analysis (Figure S11 and Figure S12). In contrast to the main CBCL brain analysis, the PLS analysis identified higher CBCL scores (i.e., greater psychopathology problems) linked to lower cortical thickness across nearly all regions (Figure S10). However, none of the LVs extracted from the elevated-CBCL sample from both PLS and CCA were statistically significant or reproducible. In the sample with full CBCL subscale endorsement (*N* = 5,196), the relationships between brain and behavior variables were similar to that of the main CBCL brain analysis but were not statistically significant or reproducible (singular values of LV_1_: PLS = .38, CCA = .18; Figure S13 and Table S5). The results of the clinical brain and cognitive brain analyses found that the distributional properties and correlational structure of the additional phenotypic measures assessed influences the reproducibility of CCA- and PLS-derived relationships, consistent with the main results (Supporting Information Section 3.6, Figures S21–S24).

## DISCUSSION

This study aimed to understand factors that influence between-method (i.e., CCA vs. PLS) and within-method (i.e., reproducibility/reliability/stability) generalizability of brain-behavior relationships. In the CBCL brain analyses, the results indicated that LV_1_ identified different multivariate patterns of brain-behavior associations using CCA or PLS, and neither CCA nor PLS LVs were consistently stable or reproducible, suggesting low between- and within-method generalizability. The NIH brain analyses identified a similar LV_1_ in PLS and CCA, which were reproducible, reliable, and stable, suggesting both between- and within-method generalizability.

### Low Between-Method Generalizability for CBCL Brain Analyses

In the CBCL brain analysis, the largest contributing variables in LV_1_ differed between CCA and PLS. CCA highlighted the relationship between social problems and the right superior temporal gyrus, whereas PLS highlighted the relationship between aggressive problems and the right pars triangularis. LV_1_ derived from CCA showed covariation (i.e., positive and negative loadings) in both CBCL scores and cortical thickness. The pattern of covariation is consistent with prior work finding covariation between brain and various phenotypic measures in a large community-derived sample using CCA (Smith et al., 2015). CCA may be more likely to identify prominent patterns of covariation between two data matrices due to the adjustment of the within-block correlation matrices in the cross-product matrix (**Ω**) submitted to the SVD. The adjustment removes the within-block variance, thereby optimizing for uniqueness. LV_1_ derived from PLS, in contrast, identified lower CBCL scores linked to mainly lower cortical thickness. The limited covariation in LV_1_ (i.e., most elements loaded in the same direction) from PLS may be due to the optimization of redundant relationships given that the cross-product matrix does not adjust for within-block correlations, a trend found in prior reports (Itahashi et al., 2020). Notably, the CBCL brain PLS LV_1_ contrasts with prior univariate analyses reporting negative associations between CBCL and cortical thickness (Ameis et al., 2014; Ducharme et al., 2011, 2014; Jacobs et al., 2021; Vijayakumar et al., 2017). However, in the elevated-CBCL sample, we found that LV_1_ in PLS linked higher behavioral scores to lower cortical thickness. Although the results were not significant, the increased correlations in the cross-product matrix (Figure S11) suggest that a larger sample might produce significant results. The contrast of these two results suggest that brain-behavior relationships identified using the CBCL at the population level may be washing out brain-behavior patterns present in children with more pronounced behavioral problems.

### Low Within-Method Generalizability for CBCL Brain Analyses

In the CBCL brain analysis, there were no consistent reproducible or reliable LVs as assessed by split-half or train-test resampling, respectively. The behavioral loading in LV_2_ was reproducible when implementing PLS. The singular value for LV_1_ in CCA was found to be reliable, a result indicating a robust, albeit small, signal greater than chance (McIntosh & Lobaugh, 2004). Furthermore, there were stable CBCL and cortical thickness elements in LV_1_ using PLS, but not CCA (see Figure S8)—an effect confirming that PLS is better suited to find stable elements when within-block correlations are moderate-to-high (McIntosh, 2021). Despite limitations in between- and within-generalizability, LV_1_ was robust (Huber, 2011) given that the sensitivity and post hoc analysis in the subsample with full subscale endorsement did not substantially alter the relationship identified for CCA or PLS. The statistical significance of LV_1_ indicates that the results are meaningful to the present sample; however, the lack of reproducibility and reliability suggests results may not be generalizable to other samples.

### High Between- and Within-Method Generalizability in the NIH Brain Analysis

In the NIH brain analysis, LV_1_ derived from CCA and PLS both linked decreased cognitive performance scores to covariation of cortical thickness. This similarity may be due to the stronger between-block correlations in the cross-product matrix (**R**_**XY**_ and **Ω**; Figure S10) compared to the CBCL brain analysis, leading the NIH scores to exhibit the same directional relationship to cortical thickness for CCA and PLS. Both the behavioral and brain elements that contributed most to LV_1_ were similar between CCA and PLS (Figure S9). The reliability and reproducibility of LV_1_ suggest that the results found are meaningful to the current sample and generalizable to other samples. Thus, between-method and within-method generalizability suggest the NIH Cognitive Toolbox may be better suited to delineate relationships between behavioral phenotypes and brain morphometry in normative population-based samples.

The findings of the main analyses in this study show consistencies with prior work. Prior work using the ABCD dataset found that cognitive performance is more predictive of brain-based measures compared to behavioral measures (Ooi et al., 2022). Another study using CCA to delineate brain-phenotype relationships found a significant latent relationship emphasizing NIH Toolbox estimated fluid intelligence and resting-state functional connectivity in default mode network (DMN) (Goyal et al., 2022). These results overlap with NIH brain LV_1_ results showing a relationship between the list sorting working memory task and structural regions of the DMN (see Figure S9). The similarities between the current study and prior work suggests that generalizable relationships within a given study may be more likely to be replicated across studies.

### Skewed Data and Low Correlations May Reduce Between- and Within-Method Generalizability

In the CBCL brain analysis, the lack of between- and within-method generalizability may have been driven by the low between-block correlations (i.e., minimal effect size, Figure S12), and the skew of the CBCL variables (Figure S14), which suggests the variance of interest is present within a small subsample with the most extreme behavioral problems. As CCA and PLS optimize for linear relationships, non-Gaussian distributions may lead to unstable results. In contrast, NIH Toolbox measures were more normally distributed (Figure S14) and featured stronger correlations between the brain and behavioral variable sets (Figure S15), a pattern likely contributing to increased between- and within-method generalizability. An additional configuration potentially driving the limited generalizability is the more compressed variance structure of the CBCL compared to NIH Toolbox scores (CBCL: 6/11 (54.5%), and NIH: 5/7 (71%) of the components were needed to explain 90% of the variance when submitted to a principal components analysis). Notably, it is unlikely that the degree of collinearity of the CBCL matrix drove the instability of CCA models, given that the NIH performance scores exhibit even higher collinearity (Figures S12 and S15). It is also unlikely that differences between continuous versus ordinal variables was driving the differences in generalizability found in the NIH brain versus CBCL brain analyses (Supporting Information Section 3.4; Figures S17–S19).

Our results suggest that generalizability of CCA and PLS are impacted by the variance structure of the phenotypic measure used when examining brain-behavior relationships. Although we did not find major differences between CCA and PLS in the NIH brain analysis, these two approaches do have different underlying philosophies that are important when considering which model to implement. The removal of the within-block correlations allows CCA to amplify the specific and unique variable(s) that most strongly characterizes the **Ω**, while PLS takes advantage of the redundancy of the within-block correlations and amplifies the collection of variables that most strongly characterizes **R**_**XY**_. Importantly, the one-to-one comparison between CCA and PLS was possible in this study because both analyses used region of interest brain data. Given that CCA maximizes for correlations, multicollinearity within a variable set will result in unstable LVs with CCA (e.g., if using voxel-wise data; Mihalik et al., 2022). In such cases, PLS should be used.

Dimensionality reduction has been used as a preprocessing step performed prior to CCA, particularly when a matrix is rank-deficient to make one or both matrices orthogonal, which eliminates multicollinearity and can improve stability of CCA solutions (Helmer et al., 2024; Mihalik et al., 2022). We did not perform a dimensionality reduction step in the current study as this would undermine one of the critical differences between CCA and PLS: the normalization of the cross-product matrix by the within-block matrices in CCA, but not PLS. This dimensionality reduction step alters the statistical properties of the maximization problem of CCA and PLS. The LVs will no longer maximize the association between the two sets of variables, but a scaled association that also factors in the within-block correlations. As a result, we decided to employ classical CCA and PLS in the current study given the use of low-dimensional matrices.

### Limitations

There are some limitations to consider when interpreting the results of this study. First, we analyzed the baseline data from ABCD, which includes children aged 9–11, a sample which may not feature substantial developmental variability of cortical thickness to facilitate strong relationships linked to parent-reported behavioral phenotypes (see Supporting Information Section 3.5 for more details). Second, we focused our analysis on classical CCA and PLS as opposed to more recent derivative approaches, such as kernel CCA or sparse PLS, which add a penalty factor to reduce the weight of variables that have a weak contribution to an LV (Mihalik et al., 2019; Witten & Tibshirani, 2009). These approaches have typically been used in datasets that include a disproportionate ratio of participants to variables (Mihalik et al., 2019), which was not the case in the current sample. Third, while it is possible that linking ordinal (CBCL scores) and continuous (cortical thickness) variables within a multivariate model may limit generalizability, a supplementary post hoc analysis suggests this is unlikely (see Supporting Information Section 3.4). Fourth, there are several ways to assess generalizability of brain-behavior relationships. While the current study capitalized on resampling statistics, alternative methods such as cross-validation approaches (Kriegeskorte, 2015) could have also been used. Fifth, while we residualized sites across all analyses, we did not group by site in the resampling analyses. However, the specific preprocessing steps are unlikely to influence the differences between the methods, as long as assumptions are not violated.

### Conclusion

Clinical neuroscience research is going through a translational crisis largely due to the challenges of delineating brain-phenotype relationships that are consistent across studies, meaningful, and generalizable (Nour et al., 2022). The results of the current study suggest that between- and within-method generalizability among CCA and PLS is influenced by sample/measurement characteristics. Low correlations between brain and behavioral measures coupled with skewed distributions likely reduces generalizability of CCA and PLS models. The results of this study suggest that the properties of the measures inputted into CCA or PLS models play a more substantial role in the generalizability of the results compared to the specific approach applied (i.e., CCA or PLS). There are several avenues of future work that should be examined to improve brain-behavior relationship delineation at the population level. First, clustering methods may be more optimal to implement when clinical scales are used as the phenotypic measure, as opposed to methods that identify a single linear dimension. Second, exploring longitudinal brain-behavior relationships using multivariate methods is important to identify within-person stability and reproducibility. Third, it is possible that using functional MRI (fMRI) acquisitions may result in greater generalizability of brain-behavior relationships given the increased variability of fMRI metrics compared to structural MRI metrics. Lastly, future work should focus on collecting larger samples of participants with greater clinical impairments and/or developing of phenotypic measures that adequately capture the variation of psychopathology at the population level (Alexander et al., 2020; Nikolaidis et al., 2022).

## ACKNOWLEDGMENTS

Data used in the preparation of this article were obtained from the Adolescent Brain Cognitive Development (ABCD) Study (https://abcdstudy.org), held in the NIMH Data Archive (NDA). This is a multisite, longitudinal study designed to recruit more than 10,000 children ages 9–10 and follow them over 10 years into early adulthood. The ABCD Study is supported by the National Institutes of Health and additional federal partners under award numbers U01DA041048, U01DA050989, U01DA051016, U01DA041022, U01DA051018, U01DA051037, U01DA050987, U01DA041174, U01DA041106, U01DA041117, U01DA041028, U01DA041134, U01DA050988, U01DA051039, U01DA041156, U01DA041025, U01DA041120, U01DA051038, U01DA041148, U01DA041093, U01DA041089, U24DA041123, U24DA041147. A full list of supporters is available at https://abcdstudy.org/federal-partners.html. A listing of participating sites and a complete listing of the study investigators can be found at https://abcdstudy.org/consortiummembers/. ABCD consortium investigators designed and implemented the study and/or provided data but did not necessarily participate in analysis or writing of this report. This manuscript reflects the views of the authors and may not reflect the opinions or views of the NIH or ABCD consortium investigators. The ABCD data repository grows and changes over time. The ABCD data used in this report came from NDA Release 4.0 (DOI:10.15154/1523041).

## SUPPORTING INFORMATION

Supporting information for this article is available at https://doi.org/10.1162/netn_a_00363.

## AUTHOR CONTRIBUTIONS

Hajer Nakua: Conceptualization; Data curation; Formal analysis; Methodology; Visualization; Writing – original draft; Writing – review & editing. Ju-Chi Yu: Visualization; Writing – review & editing. Herve Abdi: Methodology; Writing – review & editing. Colin Hawco: Conceptualization; Writing – review & editing. Aristotle Voineskos: Resources; Writing – review & editing. Sean Hill: Supervision; Writing – review & editing. Meng-Chuan Lai: Methodology; Writing – review & editing. Anne L. Wheeler: Methodology; Writing – review & editing. Anthony Randal McIntosh: Conceptualization; Formal analysis; Methodology; Supervision; Writing – review & editing. Stephanie Ameis: Conceptualization; Supervision; Writing – review & editing.

## FUNDING INFORMATION

Hajer Nakua, Centre for Addiction and Mental Health Foundation (https://dx.doi.org/10.13039/100014405). Hajer Nakua, Canadian Institutes of Health Research Doctoral Award. Colin Hawco, the National Institute of Mental Health. Colin Hawco, Centre for Addiction and Mental Health Foundation (https://dx.doi.org/10.13039/100014405). Colin Hawco, Natural Sciences and Engineering Research Council of Canada (https://dx.doi.org/10.13039/501100000038). Aristotle Voineskos, National Institute of Mental Health (https://dx.doi.org/10.13039/100000025). Aristotle Voineskos, Canadian Institutes of Health Research (https://dx.doi.org/10.13039/501100000024). Aristotle Voineskos, Canada Foundation for Innovation (https://dx.doi.org/10.13039/501100001805). Aristotle Voineskos, Centre for Addiction and Mental Health Foundation (https://dx.doi.org/10.13039/100014405). Meng-Chuan Lai, Canadian Institutes of Health Research (https://dx.doi.org/10.13039/501100000024). Meng-Chuan Lai, Centre for Addiction and Mental Health Foundation (https://dx.doi.org/10.13039/100014405). Anne L. Wheeler, Canadian Institutes of Health Research (https://dx.doi.org/10.13039/501100000024). Anne L. Wheeler, Brain Canada Foundation. Anne L. Wheeler, Natural Sciences and Engineering Research Council of Canada (https://dx.doi.org/10.13039/501100000038). Anthony Randal McIntosh, Natural Sciences and Engineering Research Council of Canada (https://dx.doi.org/10.13039/501100000038). Anthony Randal McIntosh, Canadian Institutes of Health Research (https://dx.doi.org/10.13039/501100000024). Stephanie Ameis, Centre for Addiction and Mental Health Foundation (https://dx.doi.org/10.13039/100014405). Stephanie Ameis, Canadian Institutes of Health Research (https://dx.doi.org/10.13039/501100000024). Stephanie Ameis, Tier 2 Canada Research Chair in Neuroimaging of Autism and Mental Health in Youth (https://www.chairs-chaires.gc.ca/chairholders-titulaires/profile-eng.aspx?profileId=5617).

## Supplementary Material



## TECHNICAL TERMS

Canonical correlations analysis (CCA): A multivariate technique that extracts latent variables that capture the maximum correlation between two sets of variables.
Partial least squares (PLS): A multivariate technique that extracts latent variables that captures the maximum covariance between two sets of variables.
Latent variable (LV): Composite of empirical measures that defines an underlying dimension capturing optimal linear associations between two sets of variables (e.g., brain and behavioral measures).
Between-method generalizability: When two analytical approaches show similar results (e.g., similar latent variables).
Within-method generalizability: When a given analytical approach shows consistent reproducible and reliable results.
Split-half analysis: A resampling procedure that iteratively divides the dataset into halves to determine the reproducibility of results by assessing the distribution of similarities between the two halves.
Train-test analysis: A resampling procedure that determines generalizability by iteratively using the output of 80% of the sample to predict the output from the remaining 20%.
Singular value decomposition (SVD): A matrix factorization technique that decomposes a matrix into two orthonormal singular vector matrices and a diagonal matrix of singular values.
Permutation test: A nonparametric statistical procedure that determines the significance of a statistic by generating a null distribution from the observed data.
Bootstrap analysis: A resampling-with-replacement procedure used to estimate the stability of a parameter of interest under the assumption that the data are a representative sample from a population of interest.
