# Supplementary material for: Comparing the stability and reproducibility of brain-behavior relationships found using canonical correlation analysis and partial least squares within the ABCD sample
Source: Netw Neurosci. 2024 Jul 1;8(2):576–96. doi: 10.1162/netn_a_00363 (PMC11168718; doi:10.1162/netn_a_00363)
Supplement: Supplementary file 1 [file netn-8-2-576-s001.pdf]

Table of Contents

1. Explanation of CCA and PLS ……………………………………………….2
2. Analytical Decisions …………………………………………………………5
3. Sensitivity Analyses …………………………………………………………6
4. Exploratory Analyses ………………………………………………………..13
5. Figures ………………………………………………………………………14
6. Tables ………………………………………………………………………..38
7. References …………………………………………………………………..45

1. Explanation of Canonical Correlations Analysis (CCA) and Partial Least Squares Correlations (PLS)

Both CCA and PLS approaches aim to address a maximization problem: searching for largest linear relationships between two data matrices (e.g., **X** and **Y**) based on specific constraints. The maximization problem is solved using the singular value decomposition (SVD) which is a technique that decomposes and extracts underlying dimensions (called *latent variables*) from a rectangular matrix. In PLS and CCA, the SVD decomposes the following matrices:

PLS:

$$\mathbf{R_{XY}} = \mathbf{X}^{\mathsf{T}}\mathbf{Y} \text{ (i.e., the cross-product matrix),}$$

CCA:

$$\mathbf{\Omega} = (\mathbf{X}^{\mathsf{T}}\mathbf{X})^{-0.5}\, \mathbf{X}^{\mathsf{T}}\mathbf{Y}(\mathbf{Y}^{\mathsf{T}}\mathbf{Y})^{-0.5}.$$

The SVD solves the following maximization problem for each latent variable (denoted by ℓ):

PLS:

$$s_\ell = \arg\max_{u_\ell v_\ell} \{\mathbf{u}_\ell^{\mathsf{T}}\mathbf{R_{XY}}\mathbf{v}_\ell\} \quad \text{such that} \quad \mathbf{u}_\ell^{\mathsf{T}}\mathbf{u}_\ell = \mathbf{v}_\ell^{\mathsf{T}}\mathbf{v}_\ell = 1.$$

CCA:

$$s_\ell = \arg\max_{\mathbf{u}_\ell \mathbf{v}_\ell} \{\mathbf{u}_\ell^{\mathsf{T}}\mathbf{\Omega}\mathbf{v}_\ell\} \quad \text{such that} \quad \mathbf{u}_\ell^{\mathsf{T}}\mathbf{u}_\ell = \mathbf{v}_\ell^{\mathsf{T}}\mathbf{v}_\ell = 1.$$

Note: **u** *and* **v** *are the left and the right singular vectors, and* **s** *are the singular value of the decomposed matrix (where* $s_1 \geq s_2 \geq \cdots \geq s_\ell \geq \cdots \geq s_L$*). The superscript* ^{T} *indicates the transpose of a matrix or a vector such that rows become columns.*

The maximization problem in CCA and PLS can be solved iteratively by a gradient descent algorithm such that the first pair of singular vectors identified ($\mathbf{u}_1$ and $\mathbf{v}_1$) explain the maximum amount of variance in $\mathbf{R_{XY}}$ or $\mathbf{\Omega}$. In this way, the $\mathbf{u}_1$ and $\mathbf{v}_1$ singular vectors store the coefficients of each variable (analogous to beta weights in a linear regression), respectively of **X** and **Y**, that form the first latent variable. These coefficients are called *loadings*. The singular value s_1 gives the standard deviation, therefore quantifies the variance, of the first latent variable. The second pair of singular vectors ($\mathbf{u}_2$ and $\mathbf{v}_2$) and singular value (s_2) are identified to be orthogonal to the first pair (i.e., $\mathbf{u}_1^{\mathsf{T}}\mathbf{u}_2 = \mathbf{v}_1^{\mathsf{T}}\mathbf{v}_2 = 0$) and explain the maximum amount of the remaining variance from the first latent variable with $s_1 \geq s_2$. This process continues until all

variance of $\mathbf{R_{XY}}$ or $\mathbf{\Omega}$ is explained by all latent variables. With $s_1 \geq s_2 \geq \cdots \geq s_\ell \geq \cdots \geq s_L$, the SVD ensures that the relationship between the original **X** and **Y** matrices is explained by the latent variables in a descending order with the strongest relationship (explains the greatest amount of variance) in the first latent variable.

In CCA, the singular vectors (**U** and **V**) from the SVD are further reweighted by the Cholesky decomposition of the respective within-block correlation matrix. This obtains *beta weights* which are analogous to beta weights in a linear regression. As a result, the singular values used in CCA are generalized singular values (denoted $\mathbf{U}_b$ and $\mathbf{V}_b$). The mathematical derivation of the beta weights are explained below:

$$\mathbf{SVD(\Omega) = USV^T}$$
$$\mathbf{U_b = (X^TX)^{-0.5}\ U}$$
$$\mathbf{V_b = (Y^TY)^{-0.5}\ V}$$

Formally, this decomposition can be expressed in one step as:

PLS:

$$\mathbf{R_{XY}} = \mathbf{USV}^{\mathsf{T}} \quad \text{such that} \quad \mathbf{U}^{\mathsf{T}}\mathbf{U} = \mathbf{V}^{\mathsf{T}}\mathbf{V} = \mathbf{I}$$

CCA:

$$\mathbf{\Omega} = \mathbf{U_b S V_b}^{\mathsf{T}} \quad \text{such that} \quad \mathbf{U_b}^{\mathsf{T}}(\mathbf{X}^{\mathsf{T}}\mathbf{X})\mathbf{U_b} = \mathbf{V_b}^{\mathsf{T}}(\mathbf{Y}^{\mathsf{T}}\mathbf{Y})\mathbf{V_b} = \mathbf{I},$$

Where the **U** and **V** are the matrices of the left and the right singular vectors, $\mathbf{U_b}$ and $\mathbf{V_b}$ are the matrices of the generalized left and right singular vectors, **S** is a diagonal matrix with s_ℓ on the diagonal and 0s on the off diagonal, and **I** denotes the identity matrix that has 1s on the diagonal and 0s on the off diagonal. The left (generalized) singular vector matrix **U** (and $\mathbf{U_b}$) has *n* left (generalized) singular vectors **u** (and $\mathbf{u}_b$) on the columns and describes the loadings of **X** for all latent variables. The right (generalized) singular vector matrix **V** (and $\mathbf{V_b}$) has *n* right (generalized) singular vectors **v** (and $\mathbf{v_b}$) on the columns and describes the loadings of the **Y** matrix for all latent variables. The singular values consist of the effect sizes of the multivariate relationship; in PLS, the *covariance* and, in CCA, the *correlation*. In the CCA literature, this correlation is also referred to as the canonical correlation (often denoted by δ).

The critical difference between CCA and PLS is the maximization of correlation versus covariance. This maximization occurs to the relationship between the latent scores $\mathbf{L_X}$ and $\mathbf{L_Y}$. These latent scores are projections of the matrices **X** and **Y** onto the latent dimensions by multiplying their original variables by their respective singular vectors **U** and **V** in PLS, and by their **r**espective generalized singular vectors $\mathbf{U_b}$ and $\mathbf{V_b}$ in CCA. These scores are mathematically expressed as:

PLS:

$$\mathbf{L_X} = \mathbf{XU}$$

$$\mathbf{L_Y} = \mathbf{YV}$$

$$\text{where } \mathbf{S} = \mathbf{L_X}^{\mathrm{T}}\mathbf{L_Y} = \mathbf{U}^{\mathrm{T}}(\mathbf{X}^{\mathrm{T}}\mathbf{Y})\mathbf{V};$$

$$\text{for each latent variable, } s_{\ell} = \boldsymbol{l_X}^{\mathrm{T}}\boldsymbol{l_Y}$$

CCA:

$$\mathbf{L_X} = \mathbf{XU_b}$$

$$\mathbf{L_Y} = \mathbf{YV_b}$$

$$\text{where } \mathbf{S} = (\mathbf{X}^{\mathrm{T}}\mathbf{X})^{-0.5}\, \mathbf{L_X}^{\mathrm{T}}\mathbf{L_Y}(\mathbf{Y}^{\mathrm{T}}\mathbf{Y})^{-0.5};$$

$$\text{for each latent variable, } s_{\ell} = \frac{l_X^T l_Y^T}{\sqrt{X^T X} \times \sqrt{Y^T Y}}$$

*Note: In PLS and CCA, the $\boldsymbol{L_X}$ and $\boldsymbol{L_Y}$ matrix is the product of a direct multiplication of the original scores (**X/Y**) and loadings (**U/V** or **U_b/V_b**). In PLS, the singular values in **S** are maximized by obtaining the cross-product between $\boldsymbol{L_X}$ and $\boldsymbol{L_Y}$ (i.e., covariance). In CCA, $\boldsymbol{L_X}$ and $\boldsymbol{L_Y}$ are computed the same way as in PLS; however, the singular values of the cross-product (i.e., $\boldsymbol{L_X}^T\boldsymbol{L_Y}$) are pre- and post-multiplied by the inverse of the square-root of the within-block correlations of **X** and **Y** (i.e., $(\boldsymbol{X}^T\boldsymbol{X})^{-\frac{1}{2}}$ and $(\boldsymbol{Y}^T\boldsymbol{Y})^{-\frac{1}{2}}$). This equation of computing **S** in CCA is equivalent to computing the correlation between $\boldsymbol{L_X}$ and $\boldsymbol{L_Y}$.*

1.2. Structure Coefficients for CCA

In addition to the beta weights, McIntosh et al. (2020) implemented CCA by further defining and analyzing *structure coefficients*. The structure coefficients are calculated by multiplying the within-block correlation matrices of **X** and **Y** by their respective beta weights. This step reintroduces the variance of **X** and **Y** so that the LVs generated using these structural coefficients are more similar to the LVs from PLS. The mathematical derivation of the structural coefficients are explained below:

$$\mathbf{U}_{\mathbf{structCoef}} = (\mathbf{X}^{\mathbf{T}}\mathbf{X})(\mathbf{X}^{\mathbf{T}}\mathbf{X})^{-0.5}\ \mathbf{U}_{\mathbf{b}}$$

$$\mathbf{V}_{\mathbf{structCoef}} = (\mathbf{Y}^{\mathbf{T}}\mathbf{Y})(\mathbf{Y}^{\mathbf{T}}\mathbf{Y})^{-0.5}\ \mathbf{V}_{\mathbf{b}}$$

Note: structCoef = structure coefficients

For the main paper, we interpreted the LVs generated from the beta weights across all analyses. We included the CCA results for the CBCL-brain analysis with the structural coefficients in *Figure S4*.

2. **Analytical Decisions**

2.1. Spearman versus Pearson correlation matrix

Although the main paper presented the CCA and PLS results when decomposing a Spearman cross-correlation matrix, Pearson correlations are more commonly implemented. As such, we also explored whether using a specific correlation coefficient would alter the overall results of the CBCL-brain analysis (relationship between cortical thickness and CBCL scores). We found that the identified LVs for both CCA and PLS were similar when implementing a Spearman versus a Pearson cross-correlation matrix (see *Figure 2; Figure S3*), suggesting a consistent LV for linear and monotonic relationships between **X** and **Y**. When decomposing a Pearson correlation matrix, there were 6 and 7 statistically significant LVs for the CCA and PLS models, respectively. Further, performing the split-half resampling for the PLS model using a Pearson cross-correlation matrix revealed cortical thickness loadings for LV_1 that just surpassed the reproducibility threshold (*z*-score = 2.04). This suggests that the relationships found when using a Pearson cross-product matrix may be stronger compared to using a Spearman's cross-product matrix. However, given the possibility of non-linear relationships between the cortical thickness and CBCL data, the Pearson cross-correlation may inflate the correlations. Prior work showed that using a Spearman's correlation coupled with a Fisher's *z*-transform yielded more robust results compared to using Pearson correlation coefficients on non-normal data (Myers & Sirios, 2006). As such, the relationships assessed using a Spearman's correlation are likely more accurate compared to Pearson's correlation in this sample.

2.2. Transforming the Behavioural Data

Given the skewness of the CBCL data, we attempted to transform the data to impose normality of the distribution. We used the log transform, as used in prior reports (Dienes et al. 2002; Gross et al. 2006; Tollenaar et al. 2021), however, it did not remove the skewness of the CBCL data. As a result, we decided to use the Spearman's correlation matrix (without transforming the data) to address the skewness of the CBCL scores.

2.3. Arbitrary Sign Flip Correction for Bootstrap Resampling

In the bootstrap resampling process, there is a possibility of reflections (i.e., sign flips) in the resampled matrix each time the SVD is performed. These sign flips should be corrected to reduce the estimation bias of the bootstrap resampling (McIntosh & Lobaugh, 2004). In the bootstrap resampling analysis, we generated 1000 singular vector matrices, and assessed whether the signs of the elements in each singular vector were arbitrarily flipped. This was done by multiplying each generated **U** or **V** matrix by the respective empirical **U** or **V** matrix and determining whether the diagonals of the product matrix (the product of $\mathbf{U}_{\mathbf{generated}}$ and $\mathbf{U}_{\mathbf{empirical}}$ or $\mathbf{V}_{\mathbf{generated}}$ and $\mathbf{V}_{\mathbf{empirical}}$) were negative. If the value on the diagonal was negative, then that singular vector would be multiplied by -1 to correct the sign flip. From there, we calculated the 95% confidence interval of each variable in LV_1 from the 1000 generated **U** and **V** matrices.

3. Sensitivity Analyses

3.1. Subset of sample without head injuries

To obtain this subsample, we removed participants with a "yes" (coded as 1) for the following variables: *medhx_6i* (head injury), *medhx_2c* (seizure), *medhx_2m* (multiple sclerosis), *medhx_2h* (epilepsy), *medhx_2f* (cerebral palsy), *medhx_2c* (brain injury) from the *abcd_mx01.csv* as part of the tabulated data from ABCD. Overall, for both the CBCL-brain and NIH-brain analyses, the brain-behaviour relationship identified in LV_1 when using the main sample is preserved in this subsample for both CCA and PLS suggesting that this relationship is robust against possible effects of history of brain injury (see *Figure S5*; *Figure S7*).

3.2. Subset of sample regressed for household income data

LV_1 for both CCA and PLS models in this subsample are consistent in the CBCL-brain and NIH-brain analytical sample (see *Figure S5*; *Figure S5*). This suggests that the brain-behaviour relationship identified in LV_1 for CCA and PLS is robust against variation in SES.

3.3. Including head size as a regressor

To reduce some of the multicollinearity among the cortical thickness measures, we included total brain volume as a covariate in the linear regression performed prior to conducting the CCA and PLS analyses. The use of regressors when examining cortical thickness is not consistent among prior work; some have covaried for whole brain volumetric measures in addition to age and sex (Zhu et al. 2021; Ameis et al. 2016; Hall et al. 2021), others only covary for age and sex (Owens et al. 2021), and some covary for age-squared, age x sex, age-squared x sex (Zhu et al. 2021; Modabbernia et al. 2021). Given the limited age range of the current study, we decided not to include age-squared in the models given that there was a high collinearity between age-squared and age. Further, the relationship between age and cortical thickness or psychopathology was best described by a linear model (see *Table S6* for examples). As such, for the main analyses, we added total cortical volume as a covariate in the linear regression model prior to performing the CCA or PLS. To ensure that covarying for total cortical volume was not substantially influencing the results, we compared the cross-product matrices ($\mathbf{R_{XY}}$ and $\mathbf{\Omega}$) when total cortical volume was covaried for and when it was not (but age, sex, site, and scanner were covaried for). When correlating these two matrices, we found high correlations ($r > .8$) suggesting that regressing out total cortical volume has limited influence on the subsequent analyses. See *Figure S16* for the correlation plot which depicts the Pearson correlations between $\mathbf{R_{XY}}$ and $\mathbf{\Omega}$ from the main sample when total cortical volume is and is not regressed. The diagonal of the correlation plot indicates that the majority of variables within the brain and behavioural matrix are very similar whether total cortical volume is regressed or not.

3.4. Using age/sex adjusted CBCL and NIH scores as the input behavioural matrix

To ensure that the limited between- and within-method generalizability of the CBCL-brain analysis are not driven by the ordinal nature of the unadjusted CBCL subscale scores, we conducted a series of analyses to compare results of CCA and PLS solutions when using the

normalized (or adjusted) CBCL/NIH scores (CBCLnorm, NIHnorm) and the raw CBCL/NIH scores. The normalized CBCL t-scores are adjusted based on age and sex and range from 50-100 in the current sample (with 50 indicating no endorsement of a given symptom). Following the same analytical procedure as the main CBCL/NIH-brain analysis, we performed a linear regression to remove the effects of site, scanner, and total brain volume from the normalized CBCL or NIH scores. On the brain matrix, we performed a linear regression to remove the effects of age, sex, site, scanner, and total brain volume from each variable. Following residualization, we z-transformed the data (mean centered with a standard deviation of 1). *Figure S17* depicts the distribution of the normalized CBCL and NIH scores following residualization. As can be seen, the skew of the CBCL remains prevalent even when using normalized scores.

CBCLnorm analysis: We computed the within-block matrix for the CBCLnorm matrix (see *Figure S18B)*, as well as the cross-product matrix (both $\mathbf{R_{XY}}$ and $\mathbf{\Omega}$) between the CBCLnorm and cortical thickness matrices. The results of the within-block matrix for the CBCLnorm data are qualitatively similar to that of the main CBCL-brain analysis. To quantitatively assess the similarity between using raw CBCL or normalized CBCL scores, we performed a Pearson correlation between the cross-product matrices ($\mathbf{R_{XY}}$ and $\mathbf{\Omega}$) of the CBCL-brain and CBCLnorm-brain analysis (see *Figure S18C-F*). As shown by the diagonal of the correlation plots, the cross-product matrices calculated for both PLS and CCA are highly correlated between the CBCL-brain analysis and CBCLnorm-brain analysis ($r>0.9$).

NIHnorm analysis: We conducted the same analysis using the NIH age-adjusted scores (see *Figure S19B*). In this case, the linear regression to obtain residuals following removing the effects of regressors included sex (in addition to site, scanner, and total brain volume). Similar to the results of the CBCLnorm-brain analysis, the calculated cross-block matrices when using the NIH adjusted data is very similar to that of using the uncorrected NIH scores (see *Figure S19B-F;* $r>0.9$).

Taken together, using the normalized/adjusted CBCL or NIH scores likely produces very similar outputs to the main CBCL-brain or NIH-brain analyses given the similarity of the cross-product matrices which will be inputted into the SVD. As a result, the differences found in within- and between-method generalizability in the CBCL/NIH-brain analyses is unlikely due to differences in the mathematical properties between the CBCL and NIH matrices (i.e., using an ordinal instead of continuous scale).

We also submitted the cross-product matrices ($\mathbf{R}_{XY}$ and $\mathbf{\Omega}$) from the CBCLnorm-brain and NIHnorm-brain analyses to an SVD. We found comparable singular values (LV_1 – CBCLnorm-brain singular value: PLS = 0.35, CCA = 0.13; NIHnorm-brain singular value: PLS = 0.42, CCA = 0.2) and top loadings to the main CBCL-brain and NIH-brain analyses. The PLS CBCLnorm-brain analysis emphasized the relationship between aggressive (loading = -0.44) and thought (loading = -0.4) problems with cortical thickness of the right posterior cingulate gyrus (loading = 0.28) and left parahippocampal gyrus (loading = -0.27). The CCA CBCLnorm-brain analysis emphasized the relationship between anxiety/depression symptoms (loading = -0.63) and rule-breaking behaviour (loading = 0.64) with cortical thickness in the right superior frontal gyrus (loading=l0.39) and right precentral gyrus (loading=-0.35). Both the PLS and CCA NIHnorm-brain analysis emphasized the relationship between performance on the list sorting working memory task (loading: PLS = -0.6, CCA = -0.4) and the picture vocabulary task (loading: PLS = -0.41; CCA = -0.37) problems with cortical thickness of the left pars opercularis (loading: PLS = 0.39, CCA = 0.4) and left parahippocampal gyrus (loading: PLS = -0.37, CCA = -0.33).

Finally, we performed the split-half resampling procedure for the CBCLnorm-brain and NIHnorm-brain analyses to determine whether there would be differences in the reproducibility of the loadings. As expected, given the similarities of the cross-product matrices, the results of the split-half analyses are very similar to that of the CBCL-brain and NIH-brain analyses (see *Figure S20*). One notable difference is that the LV_1 behavioural loadings in the NIHnorm-brain analysis were not found to be reproducible. Given that CCA performs more poorly when there is high collinearity within a given matrix, it is possible that the lack of reproducibility may be due to the slightly higher within-block correlations of the behavioural matrix of the NIH age-adjusted scores which may be the result of adjusting the scores by a narrow age-range (9-11 years old).

3.5. Variance structure of cortical thickness matrix

We wanted to determine the variance structure of the cortical thickness matrix as it may facilitate further interpretations of the CCA- and PLS-derived models. This may provide increased insight into the limited generalizability of the CBCL-brain analysis. To determine the variance structure of the cortical thickness matrix, we z-transformed (mean of 0, standard deviation of 1) the cortical thickness scores prior to residualization and then submitted this matrix to a principal components analysis (PCA). This analysis revealed that 44/68 (64.7%) of the components were

needed to explain 90% of the variance in the cortical thickness matrix. Additionally, only 12/68 (17.6%) of components had an eigenvalue >1 which is a common heuristic (i.e., the Guttman-Keiser approach) used to identify relevant components (Jackson, 1993). Although it is unclear whether the identified statistical variance is indicative of limited developmental variance in the sample, it is possible that the cross-product matrix of the CBCL-brain analysis does not feature enough behavioural and brain variability to capture generalizable and stable linear latent dimensions.

3.6. Post-hoc clinical-brain and cognitive-brain analyses

We implemented a third post-hoc analysis to further probe whether CCA- and PLS-derived latent relationships would be largely impacted by the scale of measurement (i.e., self/parent-report or performance based). We examined additional clinical and cognitive measures from the baseline ABCD sample to perform a post-hoc clinical-brain and cognitive-brain analysis. For the behavioural matrix of the clinical-brain post-hoc analysis, we included 4 subscale scores from the self-report scale, the Behavioral Inhibition/Behavioral Approach System (BIS/BAS; Pagliaccio et al., 2016) which captures reward responsiveness, motivation/drive, fun seeking, and overall summed score. We also included 5 subscale scores from the parent-report UPPS-P for Children's Survey (Lynam et al., 2007; Watts et al., 2020); positive urgency, negative urgency, lack of premeditation, lack of perseverance, sensation seeking. Following the removal of missing clinical data, there were 9164 participants in the clinical-brain analysis. For the behavioural matrix of the cognition-brain post-hoc analysis, we included performance scores from the 7 trials of the Rey Auditory Verbal Learning Test (RAVLT; Strauss et al., 2006) which measures verbal learning and memory using immediate and delayed recall. Following the removal of missing RAVLT data, there were 8575 participants included in the cognitive-brain post-hoc analysis. For both analyses, we used cortical thickness parcellated from the Desikan-Killiany Atlas as the brain matrix (as was used throughout the main CBCL-brain and NIH-brain analyses).

We performed 5 analyses as part of this post-hoc analytical exploration: 1) evaluated the Pearson correlation structure between all clinical (CBCL, BIS/BAS, UPPS-P) and all cognitive (NIH Toolbox, RAVLT) measures, 2) evaluated the distributions of the clinical and cognitive

scores following residualization (identical procedure of the residualization of the main CBCL-brain and NIH-brain found in the methods section), 3) calculated the cross-product matrices ($\mathbf{R_{XY}}$ and $\mathbf{\Omega}$) between the clinical-brain and cognitive-brain analysis, 4) determined the strength of the singular values of each analysis, and, 5) performed the split-half analysis to determine the reproducibility of the loadings.

Clinical-brain analysis: (1) The clinical subscale scores show a range of distributional patterns (*Figure S21*, left panel). Many of the measures show a skewed distribution, although not as skewed as the CBCL subscale distribution (*Figure S12*). Subscale measures from the UPPS-P show varied distribution, with some showing a unimodal distribution, others showing evidence of a bimodal distribution, and some showing a more normal distribution. (2) There were relatively low correlations between the CBCL scores and both the BIS/BAS and UPPS-P scores ($r < 0.2$; 0.06±0.05, *Figure S22A*). (3) See *Figure S23A* for the cross-product matrices of the clinical-brain analysis (correlation matrix range: R_{XY} = -0.04 - 0.05, Ω = -0.04 - 0.03). (4) The singular values for the clinical-brain analysis in LV_1 were similar to those found in the main CBCL brain analysis (CCA=0.13, variance explained=20%; PLS=0.26, covariance explained=51.9%). (5) The PLS-derived loadings were found to be reproducible (see *Figure S24*) for the brain and behavioural loadings for LV_1 and LV_2. The CCA-derived loadings were not found to be reproducible across any of the LVs.

Cognitive-brain analysis: (1) The trial-based scores from the RAVLT show a range of distributional patterns, in which some scores show normal distributions, whereas others show a varied and slightly skewed distribution (*Figure S21*; right panel). (2) We find moderate correlations between the RAVLT scores and NIH toolbox scores (r=0.15-0.4, 0.27±0.07, *Figure S22*; right panel). (3) See *Figure S23B* for the cross-product matrices of the cognitive-brain analysis (correlation matrix range: R_{XY} = -0.07 - 0.05, Ω = -0.04 - 0.04). (4) The singular values and explained variance for LV_1 in the cognitive-brain analysis was higher than the clinical-brain analysis (CCA=0.17, variance explained=31.5%; PLS=0.5, covariance explained=76.2%). (5) The CCA- and PLS-derived brain loadings and PLS-derived behaviour loadings for LV_1 were reproducible. The PLS-derived brain and behaviour loadings for LV_2 were reproducible (*Figure S23*).

The findings of the post-hoc analysis provide increased insight into the factors that may influence CCA- and PLS-derived brain-behaviour relationships. Overall, the results of the post-hoc analyses show similar trends to the main CBCL- and NIH-brain analyses. The cognitive-brain analysis showed stronger and more reproducible multivariate relationships (i.e., both CCA and PLS showed reproducible loadings) compared to the clinical-brain analysis. These results provide further evidence to support our conclusions that cognitive and/or performance-based measures may result in more reproducible brain-behaviour relationships when compared to clinical and self- or parent-report measures in normative samples across multivariate brain-behaviour analyses and resampling procedures.

Some of the results of the post-hoc analyses highlight notable differences from the main CBCL-brain and NIH-brain results. PLS-derived loadings of the clinical-brain analysis were found to be reproducible, in contrast with the CBCL-brain analyses. It is possible that this is due to the distribution of both BIS/BAS and UPPS-P scores being less skewed than the CBCL subscale scores. The decreased skew is likely due to the greater endorsement of symptoms across these two measures. Specifically, <10% of the participants included in the clinical-brain analysis had zero endorsement in the BIS/BAS, and <15% having the lowest score for the UPPS-P (score of 4). In contrast, there were 56.5% of the participants included in the main CBCL-brain analysis that had zero endorsement on at least one subscale. The BIS/BAS and UPPS-P capture constructs that may be more closely linked to executive functioning processes compared to psychopathology. For example, impulsivity is closely linked to impaired self-regulation, an important component of executive functioning (Fino et al., 2014; Nigg, 2017). The reproducibility of the PLS-derived loadings from the clinical-brain analysis suggests that reported measures (self or parent) could result in reproducible multivariate brain-behaviour relationships, depending on the endorsement of the constructs being measured. Importantly, the reproducible PLS-derived results compared to CCA may be due to PLS optimizing for redundant relationships (see discussion in main paper) leading to the first LV capturing the *mean* relationship between the two matrices. This configuration optimizes for higher signal-to-noise of the variance compared to CCA. It is possible that the more executive-functioning oriented constructs captured in the BIS/BAS and UPPS-P are more likely to be endorsed in a large

normative sample, leading to greater variance and possibility of finding significant results. More generally, these results may suggest that PLS may be more optimal when using clinically oriented scales that capture developmentally appropriate constructs that are widely endorsed.

In the cognitive-brain analysis, while the RAVLT trial scores capture cognitive constructs, they are not standardized and are considered to be ordinal count scores. As a result, the mathematical properties of the NIH Cognitive Toolbox Scores and the RAVLT scores differ. However, despite this difference, we find largely similar results between the NIH-brain and cognition-brain analyses. This suggests that the mathematical properties of the original variables may not largely influence the generalizability of solutions, consistent with the analysis performed in supplementary section 3.4. Instead, it is more likely that the distributional properties and correlational structure influences the degree to which CCA- and PLS-derived loadings will be reproducible. Overall, this post-hoc analysis highlights the importance of factoring in endorsement of measures when implementing multivariate statistical approaches, in particular, CCA and PLS. This post-hoc analysis provides further evidence to support that skewed (or non-normal) distributions and low cross-product correlation matrices increase the risk of low generalizability when performing CCA and PLS.

4. Exploratory analyses

4.1. Subsample with Higher Psychopathology

In the subset of the sample with an elevated CBCL total score (*t*-score > 60; *n* = 1016), the cross-block correlations in the $\mathbf{R}_{\mathbf{XY}}$ and $\mathbf{\Omega}$ matrices were larger than that of the main sample (*Figure S11*). The singular values were also higher than the main sample (LV_1: PLS = .67, CCA = .33). None of the LVs were statistically significant or reproducible across the CCA and PLS analysis.

4.2. Subsample with endorsement of all CBCL subscales

Our second post-hoc analysis examined a subset of the ABCD participants who had some endorsement of each CBCL subscale score (i.e., no value of 0; *n* = 5196). This analysis was performed to assess whether the 0-inflation of the CBCL scores was driving the low within-

method generalizability of the results. The results revealed similar brain-behaviour relationships as the main sample (singular values of LV_1: PLS = .3, CCA = .15; *Figure S13*). None of the LVs were statistically significant or reproducible.

5. Figures

Figure S1. Consort Diagram describing the number and reason of participant exclusion from the analysis.

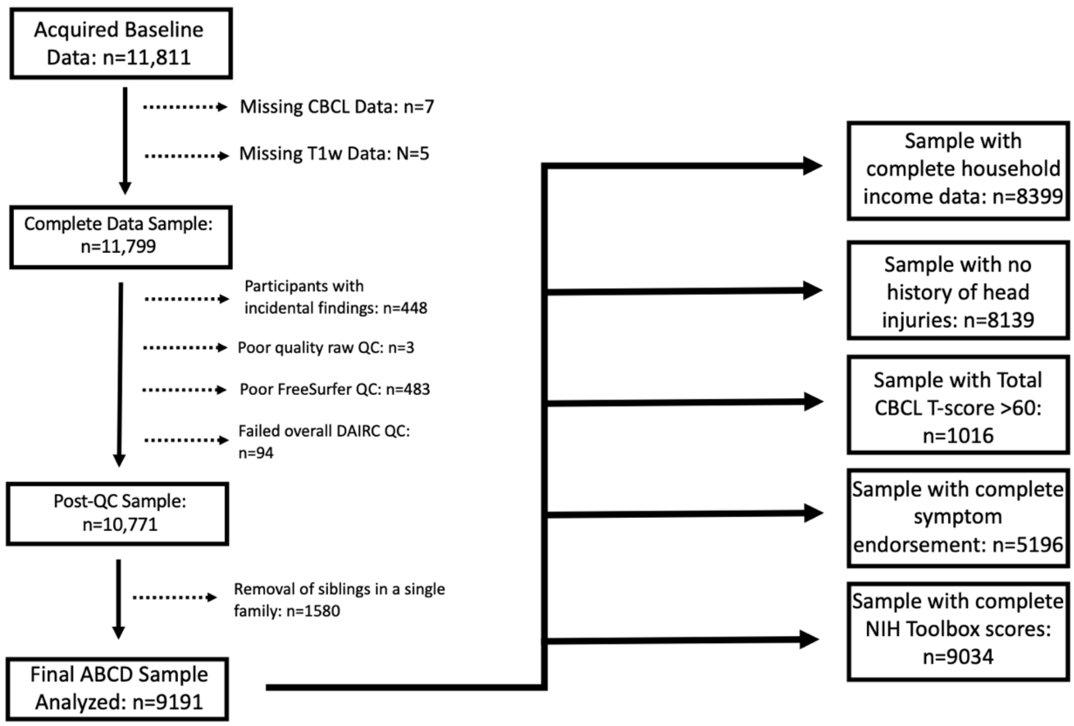

Note: The variables used to exclude participants are as follows. The incidental findings variable was *mrif_score* and participants were excluded if they were considered to "need clinical referral" or "immediate clinical referral". Participants with a zero value for the *iqc_t1_ok_ser* variable were excluded indicating that they had poor quality raw T1-weighted scans. Participants were excluded if they received a "reject" from the *fsqc_qc* variable indicating they failed FreeSurfer QC. Finally, any additional participants who failed the DAIRC QC were excluded (received a zero for the *imgincl_t1w_include* variable). The ABCD dataset includes data collected from siblings, twins and triplets as part of the sample. To reduce multicollinearity, we only retained one sibling per family.

Figure S2. Correlation plots depicting the Pearson correlations of the behavioural or brain loadings between CCA and PLS in the CBCL-brain analysis

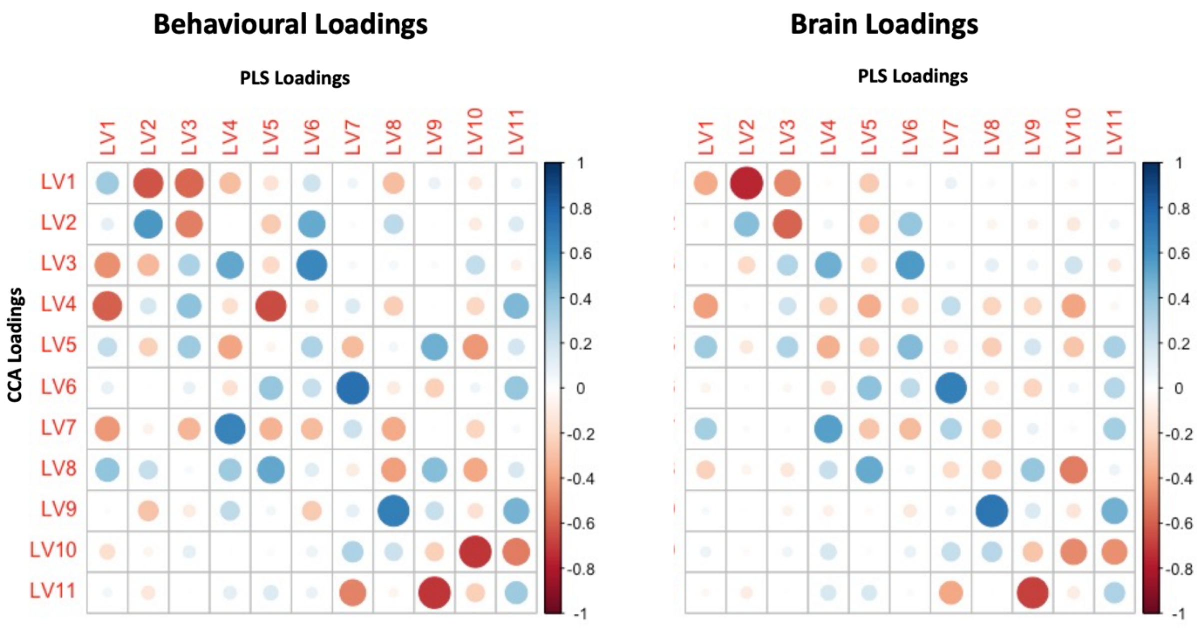

Note: This figure shows the zero-order Pearson correlation coefficients of the behavioural and brain loadings between CCA and PLS. LV = latent variable.

Figure S3. CCA and PLS results when implementing a Pearson cross-correlation matrix to examine the relationship between cortical thickness and CBCL scores.

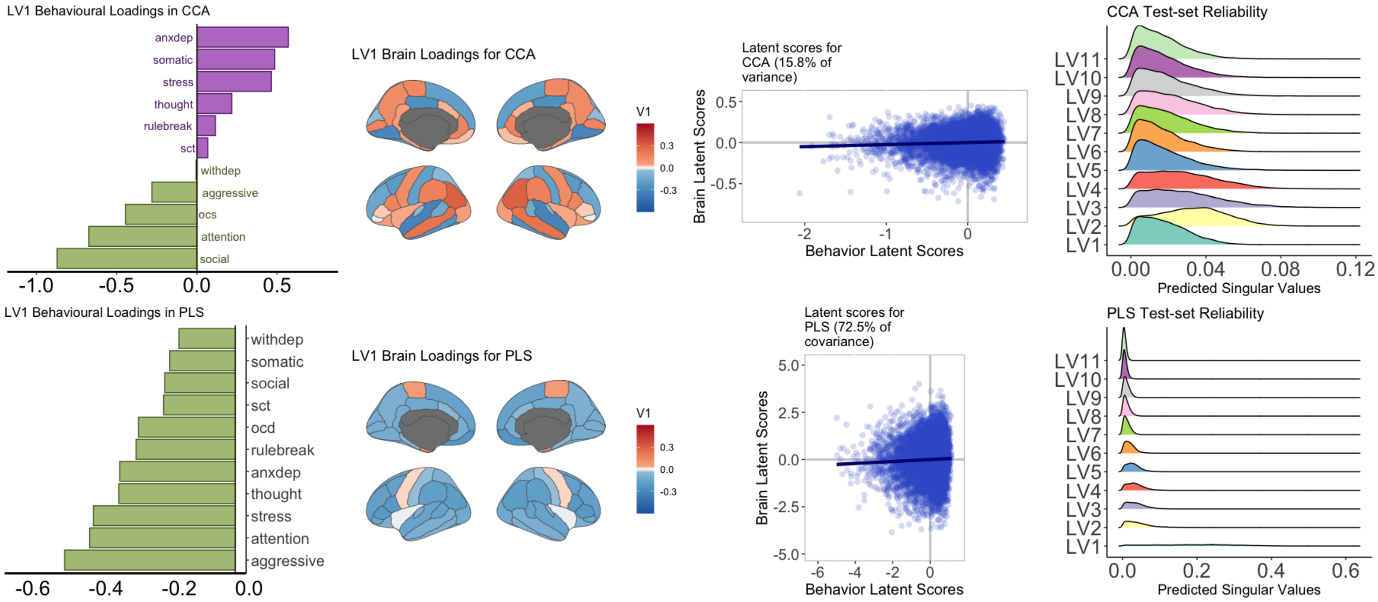

Note: Unthresholded behaviour and brain loadings from the PLS and CCA analysis performed in the main sample using a Pearson cross-correlation matrix for **Rxy** and **Ω**. Overall, the relationships in LV_1 using a Pearson cross-correlation matrix are similar to those using a Spearman cross-correlation matrix. The LVs were not stable for CCA and PLS. Prior to calculating the latent scores, the brain and behavioural loadings have been standardized by the singular values. OCD = obsessive compulsive disorder (symptoms), withdep = withdrawn/depression symptoms, sct = sluggish-cognitive-tempo, anxdep = anxiety/depression symptoms, rulebreak = rule breaking behaviour.

Figure S4. LV_1 CCA loadings for structure coefficients in the CBCL-brain analysis

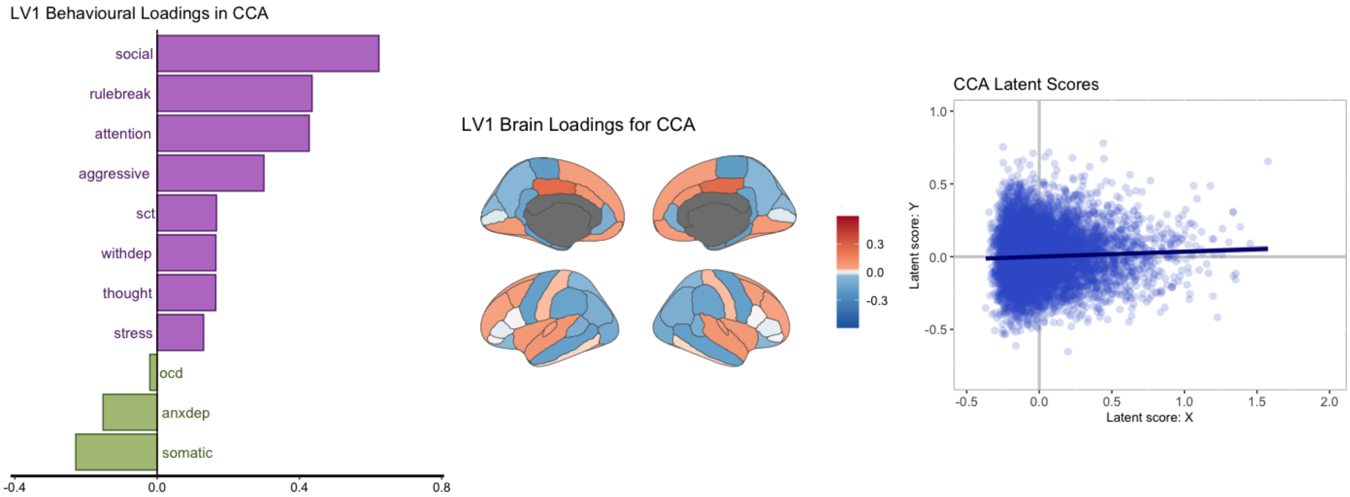

Note: A spearman cross-correlation matrix was used in this analysis. The pattern of LV_1 is similar to that when using the generalized singular vectors (i.e., loadings) from the SVD (called beta weights). Social problems remain the highest behavioural loading and we find patterns of covariation between the behaviour and brain loadings. Prior to calculating the latent scores, the brain and behavioural loadings have been standardized by the singular values. OCD = obsessive compulsive disorder (symptoms), withdep = withdrawn/depression symptoms, sct = sluggish-cognitive-tempo, anxdep = anxiety/depression symptoms, rulebreak = rule breaking behaviour. LV_1 when using the structural coefficients accounted for 19.3% of the variance.

Figure S5. CCA and PLS loadings for the two sensitivity analyses; controlling for household income (SES Subset) and removing participants without head injuries (No Head Injury Subset) in the CBCL-brain analysis

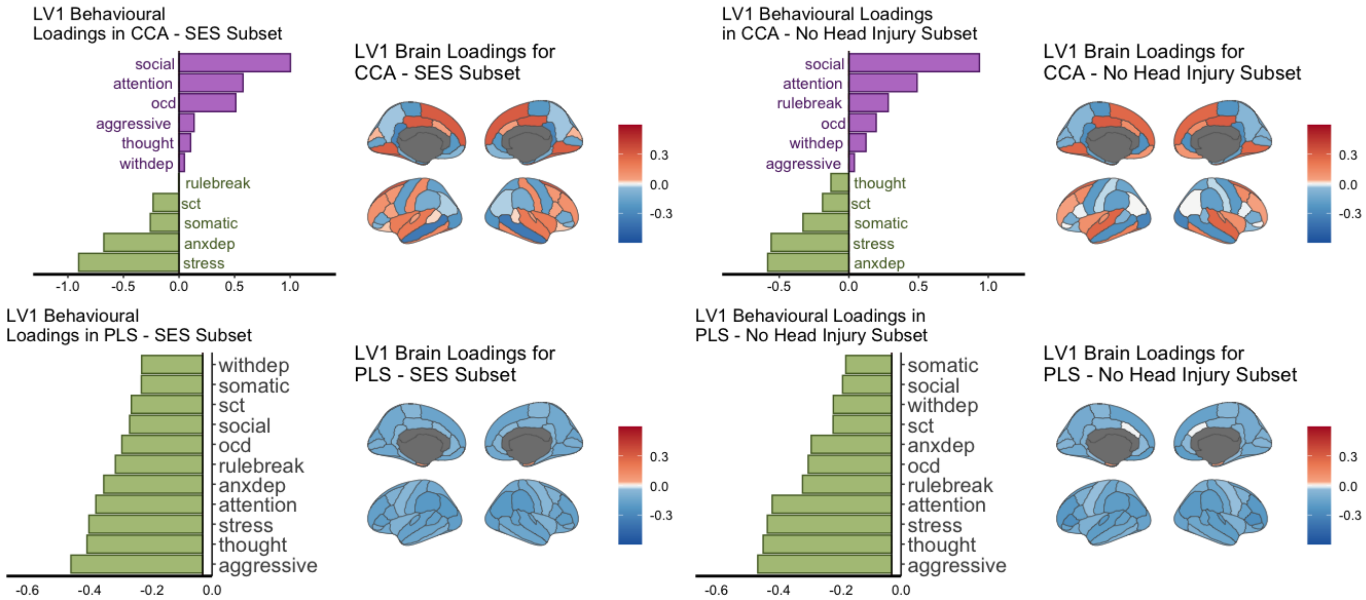

Note: In both sensitivity analysis subsets, we find similar relationships to the CBCL-brain analysis from the main manuscript. Social problems and aggressive behaviours are the highest contributing behavioural variable in LV_1 for CCA and PLS, respectively. We find overall covariation between behavioural and brain loadings in CCA (i.e., positive and negative loadings in the behavioural measures linked to positive and negative loadings in the brain measures). In the PLS analysis, we find the same trend such that decreased behavioural loadings is linked to decreased brain loadings (i.e., lower behavioural problems are linked to decreased cortical thickness). OCD = obsessive compulsive disorder (symptoms), withdep = withdrawn/depression symptoms, sct = sluggish-cognitive-tempo, anxdep = anxiety/depression symptoms, rulebreak = rule breaking behaviour.

Figure S6. Correlation plots depicting the Pearson correlations of the behavioural or brain loadings between CCA and PLS in the NIH-brain analysis

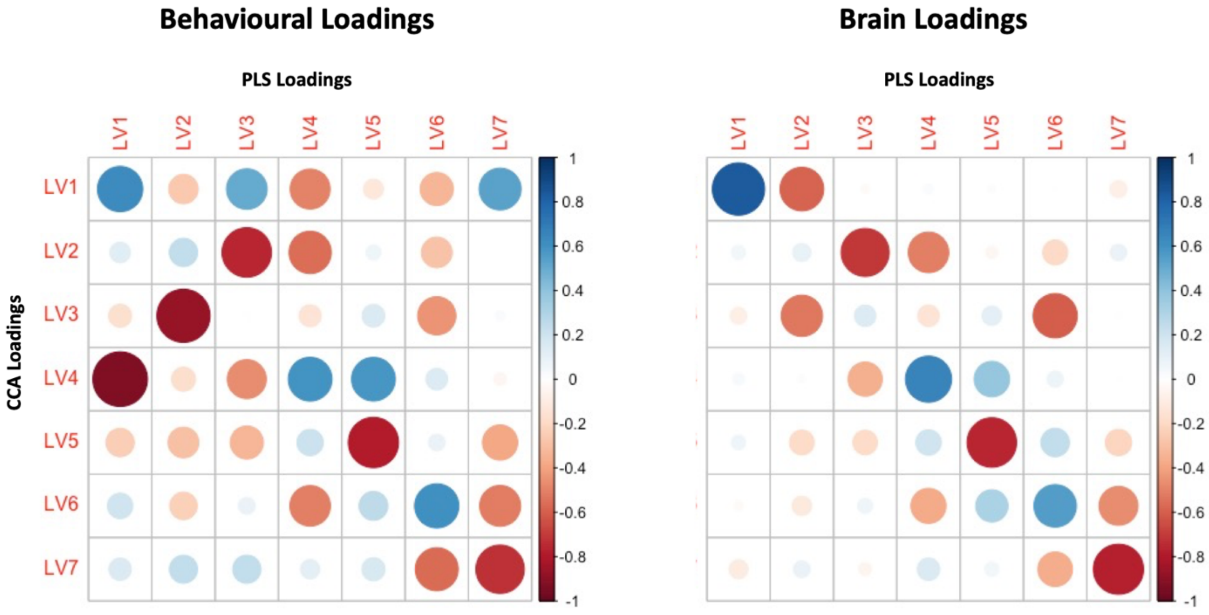

Note: This figure shows the zero-order Pearson correlation coefficients of the behavioural and brain loadings between CCA and PLS. LV = latent variable.

Figure S7. CCA and PLS analytical results for the two sensitivity analyses in the NIH-brain analysis; controlling for household income (SES Subset) and removing participants without head injuries (No Head Injury Subset).

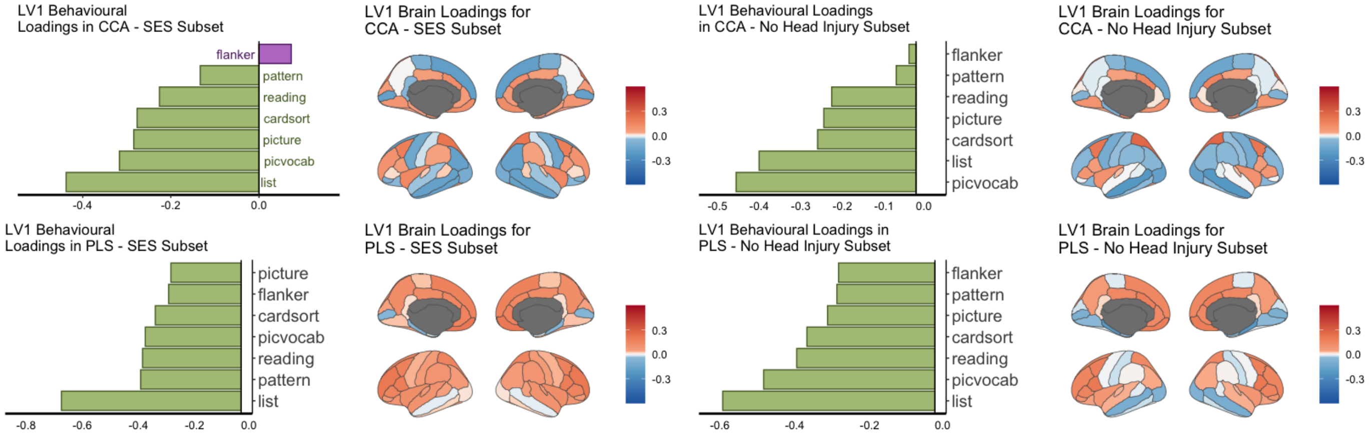

Note: In both sensitivity analysis subsets, we find overall similar relationships to the results of the NIH-brain analysis included in the main manuscript. One difference is the positive loading of the Flanker task in the CCA when regressing out household income. Performance on the list sorting working memory task is consistently the top contributing variable from the NIH scores (except in the CCA when removing participants with no head injuries). We find overall covariation in the brain loadings in CCA and PLS (i.e., positive and negative loadings), however PLS results show more positive loadings. Flanker = Flanker Task, pattern = pattern comparison processing speed task, cardsort = dimensional change card sort task, reading = oral reading recognition task, picture = picture vocabulary task, list = list sorting working memory task, picvocab = picture vocabulary task.

Figure S8. Barplots depicting the loadings of the CBCL and cortical thickness elements in LV_1 of the CBCL-brain analysis

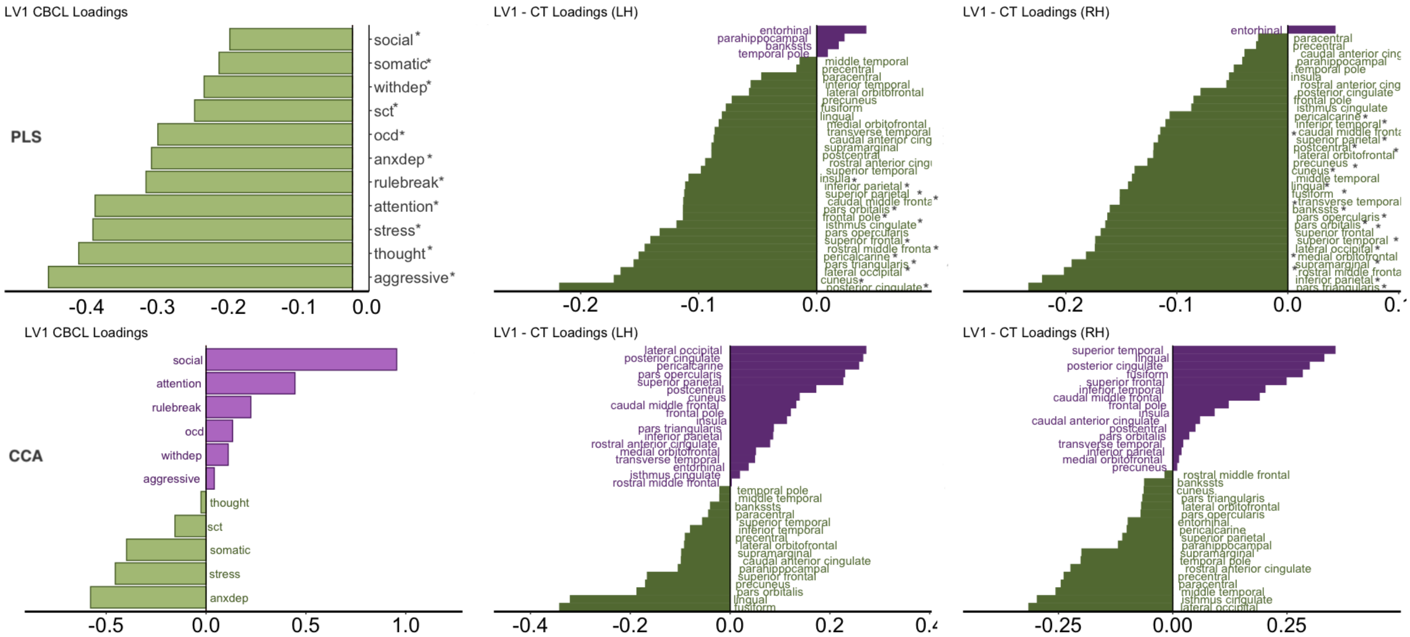

Note: Asterisks indicate that the element was stable as assessed by bootstrap resampling (i.e., the 95% confidence interval did not include zero). The asterisk may be after or before the name of the element. OCD = obsessive compulsive disorder (symptoms), withdep = withdrawn/depression symptoms, sct = sluggish-cognitive-tempo, anxdep = anxiety/depression symptoms, rulebreak = rule breaking behaviour.

Figure S9. Barplots depicting the loadings of the NIH and cortical thickness elements in LV_1 of the NIH-brain analysis.

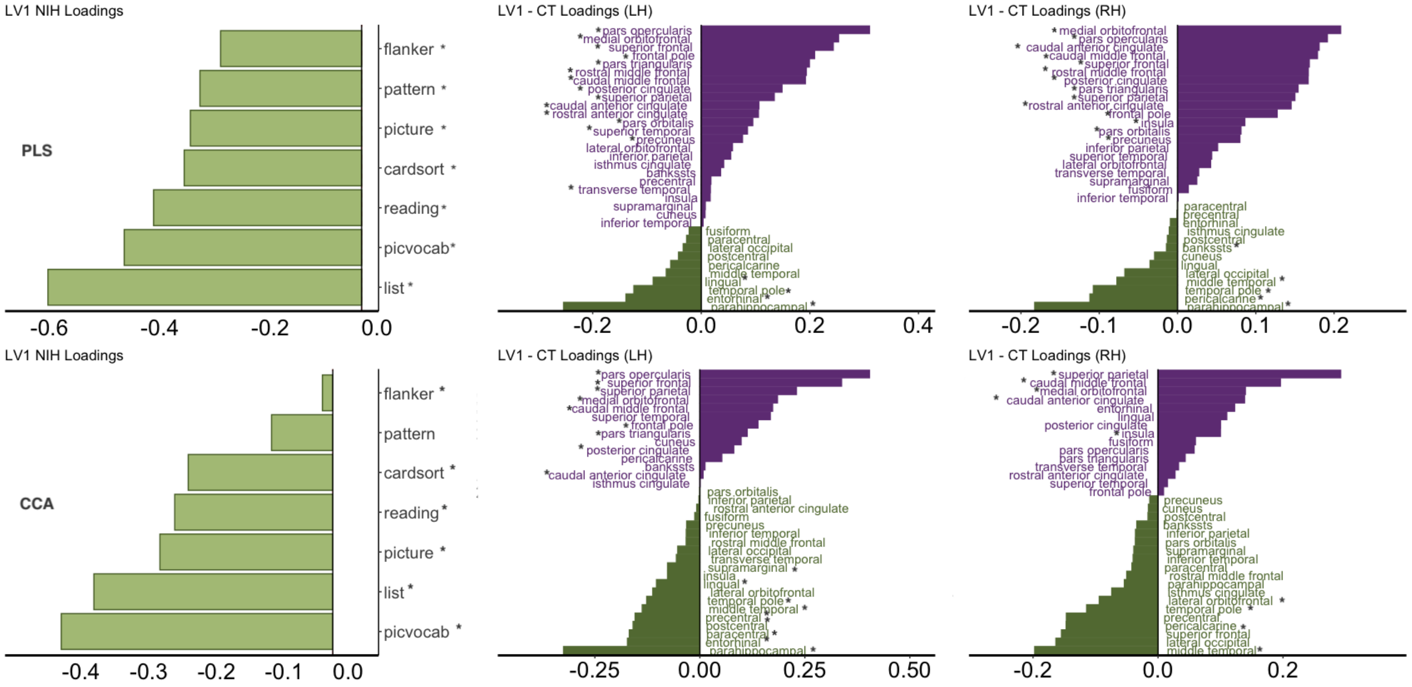

Note: Asterisks indicate that the element was stable as assessed by bootstrap resampling (i.e., the 95% confidence interval did not cross zero). The asterisk may be after or before the name of the element. Flanker = Flanker Task, pattern = pattern comparison processing speed task, cardsort = dimensional change card sort task, reading = oral reading recognition task, picture = picture vocabulary task, list = list sorting working memory task, picvocab = picture vocabulary task.

Figure S10. LV_1 CCA and PLS loadings for the elevated-CBCL subsample (CBCL t-score > 60, n = 1016).

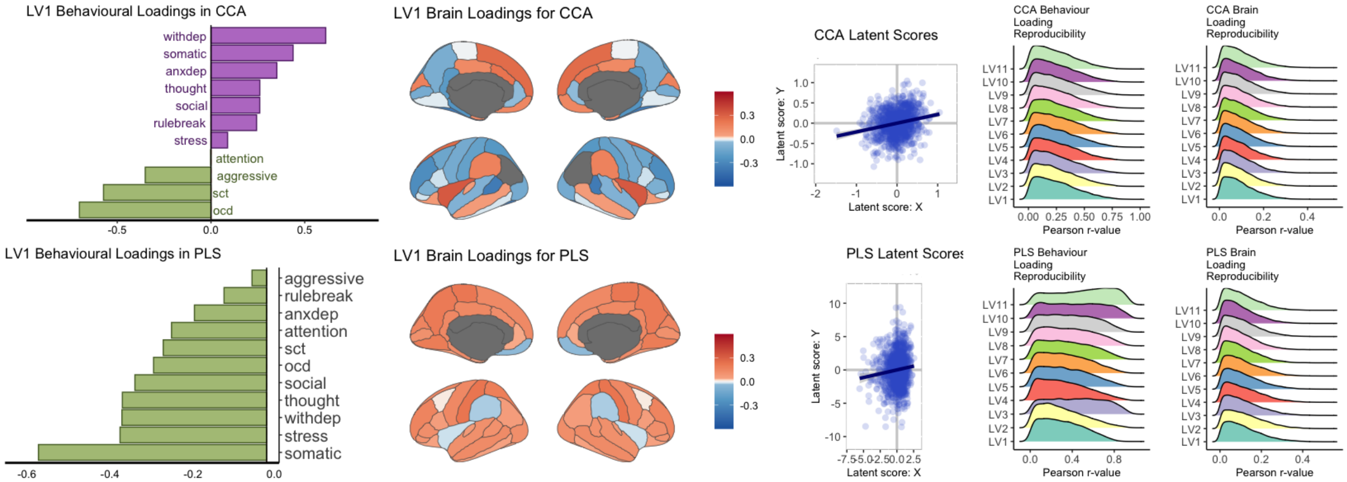

Note: Unthresholded behaviour and brain weights from the PLS and CCA analysis performed in the subsample with elevated CBCL scores. Overall, the brain-behaviour relationships in LV_1 differed between the CCA and PLS analyses in the main sample. Although the CCA analysis depicts the covariation trend found in the main sample, withdrawn/depression symptoms have the highest behavioural loading. The PLS analysis depicts a similar homogeneous relationship, however, less behavioural problems (i.e., lower CBCL scores) is linked to increased cortical thickness. Prior to calculating the latent scores, the brain and behavioural loadings have been standardized by the singular values. LV_1 for CCA accounted for 14.7% of the variance, and LV_1 for PLS accounted for 53.9% of the covariance. OCD = obsessive compulsive disorder (symptoms), withdep = withdrawn/depression symptoms, sct = sluggish-cognitive-tempo, anxdep = anxiety/depression symptoms, rulebreak = rule breaking behaviour.

Figure S11. Within- and between-block correlations of the post-hoc subsample with elevated CBCL scores (CBCL Total T-score >60; n=1016).

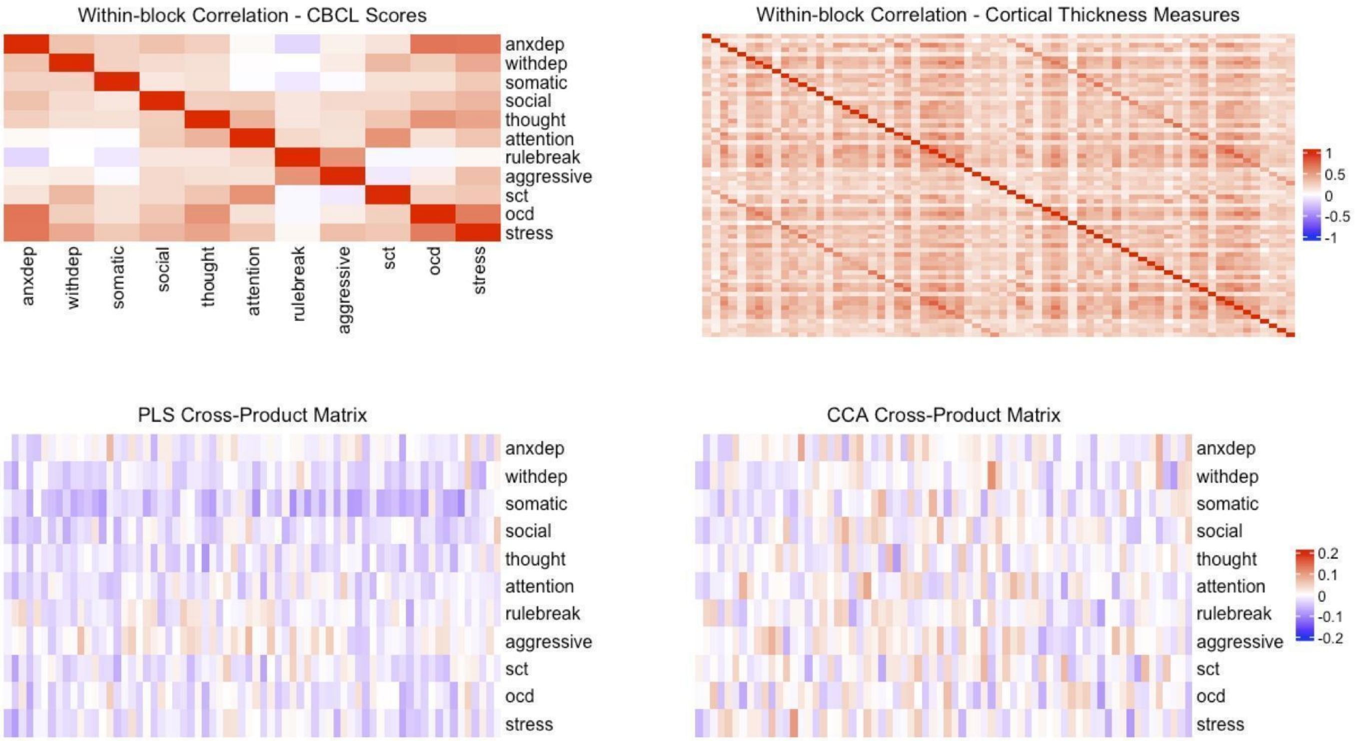

Note: The correlation figures depict zero-order Pearson correlation r-values. The X-axes labels for the cross-product matrices are the cortical thickness ROIs which were excluded from the figure for clarity of the visual representation. Similarly, the X and Y-axes labels are not included in the cortical thickness within-block correlation matrix (top right) to make the correlation matrix easier to interpret. OCD = obsessive compulsive disorder (symptoms), withdep = withdrawn/depression symptoms, sct = sluggish-cognitive-tempo, anxdep = anxiety/depression symptoms, rulebreak = rule breaking behaviour. PLS cross-product matrix = **Rxy**, CCA cross-product matrix = **Ω**.

Figure S12. Within- and between-block correlations of the CBCL-brain analysis

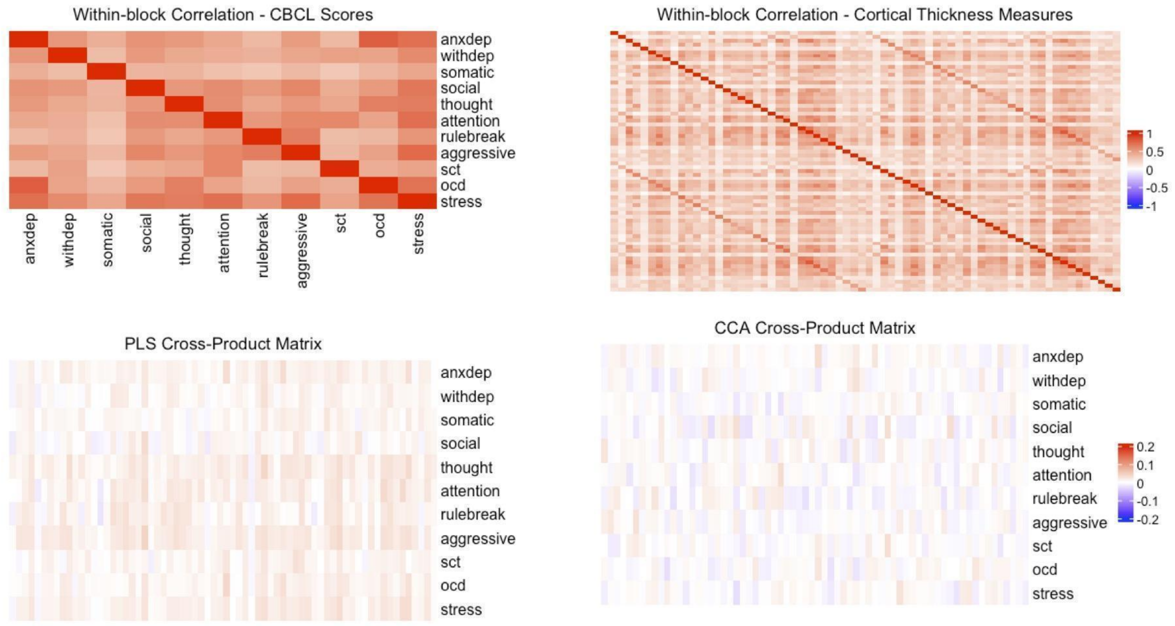

Note: The correlation figures depict zero-order Pearson correlation r-values. The X-axes labels for the cross-product matrices are the cortical thickness ROIs which were excluded from the figure for clarity of the visual representation. Similarly, the X and Y-axes labels are not included in the cortical thickness within-block correlation matrix (top right) to make the correlation matrix easier to interpret. OCD = obsessive compulsive disorder (symptoms), withdep = withdrawn/depression symptoms, sct = sluggish-cognitive-tempo, anxdep = anxiety/depression symptoms, rulebreak = rule breaking behaviour. PLS cross-product matrix = $\mathbf{R_{XY}}$, CCA cross-product matrix = $\mathbf{\Omega}$.

Figure S13. LV_1 CCA and PLS loadings for the subsample with full subscale symptom endorsement (n = 5196)

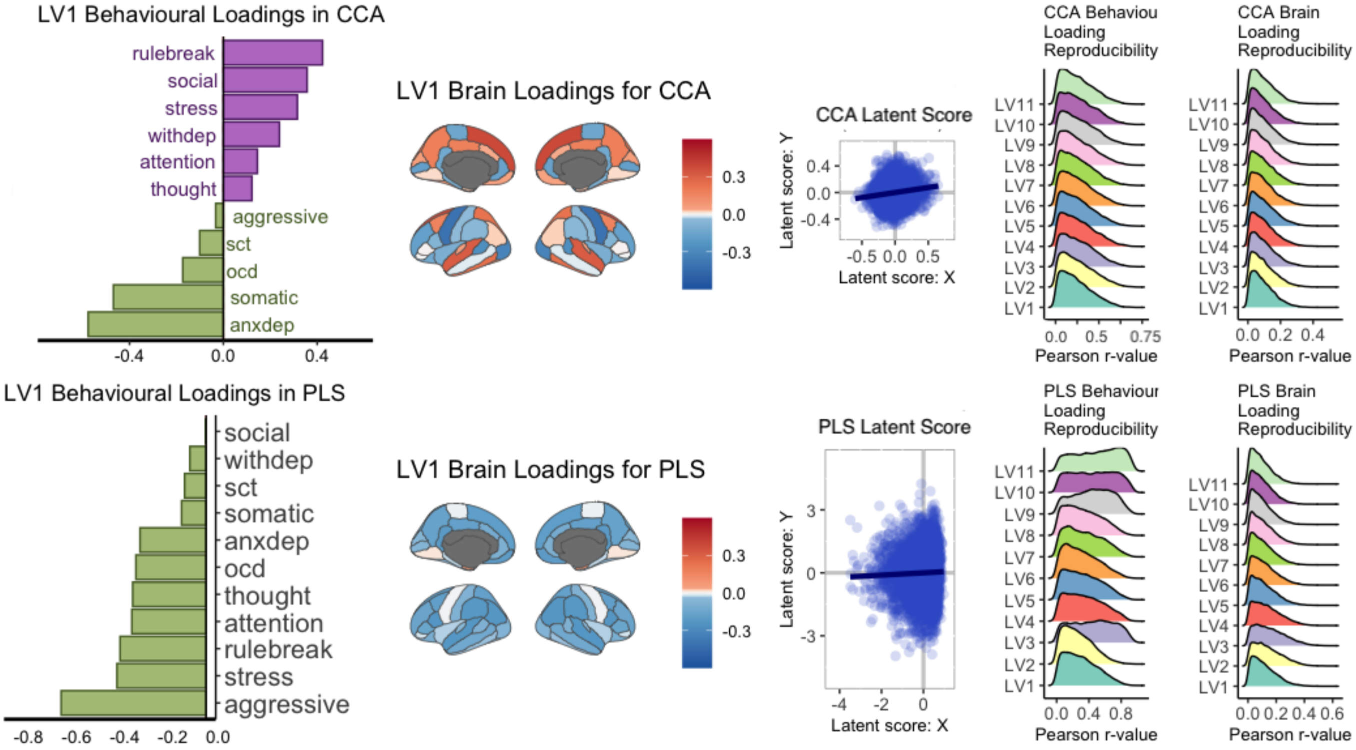

Note: Unthresholded behaviour and brain loadings from the PLS and CCA analysis performed in the subsample with subscale endorsement for each of the CBCL subscales. Overall, the brain-behaviour relationships found in LV_1 in this subsample are similar to that found in the primary analysis. Prior to calculating the latent scores, the brain and behavioural loadings have been standardized by the singular values. LV_1 for CCA accounted for 16% of the variance, and LV1 for PLS accounted for 52.3% of the covariance. OCD = obsessive compulsive disorder (symptoms), withdep = withdrawn/depression symptoms, sct = sluggish-cognitive-tempo, anxdep = anxiety/depression symptoms, rulebreak = rule breaking behaviour.

Figure S14. Distributions of residuals of the behavioural variables used in the CBCL-brain, NIH-brain, and post-hoc analyses.

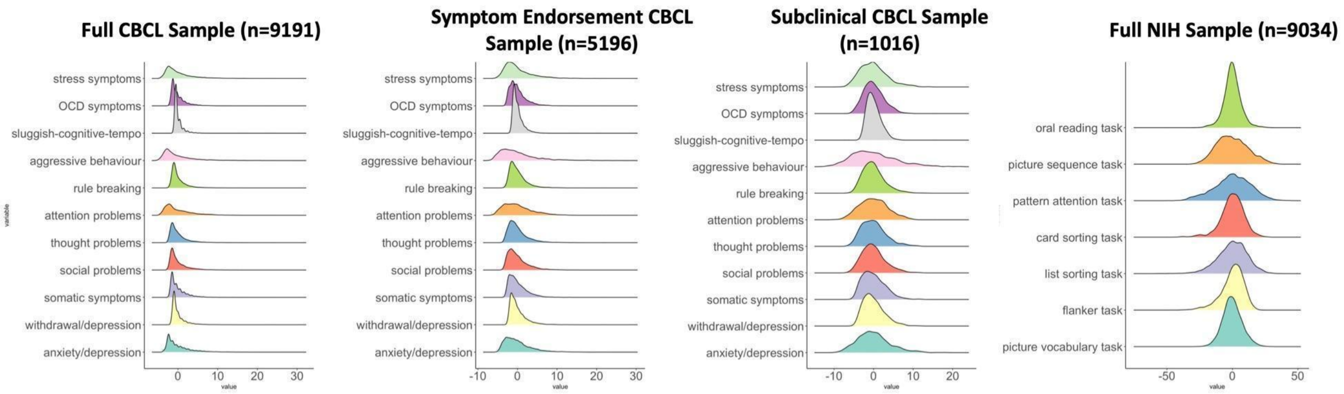

Figure S15. Within- and between-block correlations of the NIH-brain analysis

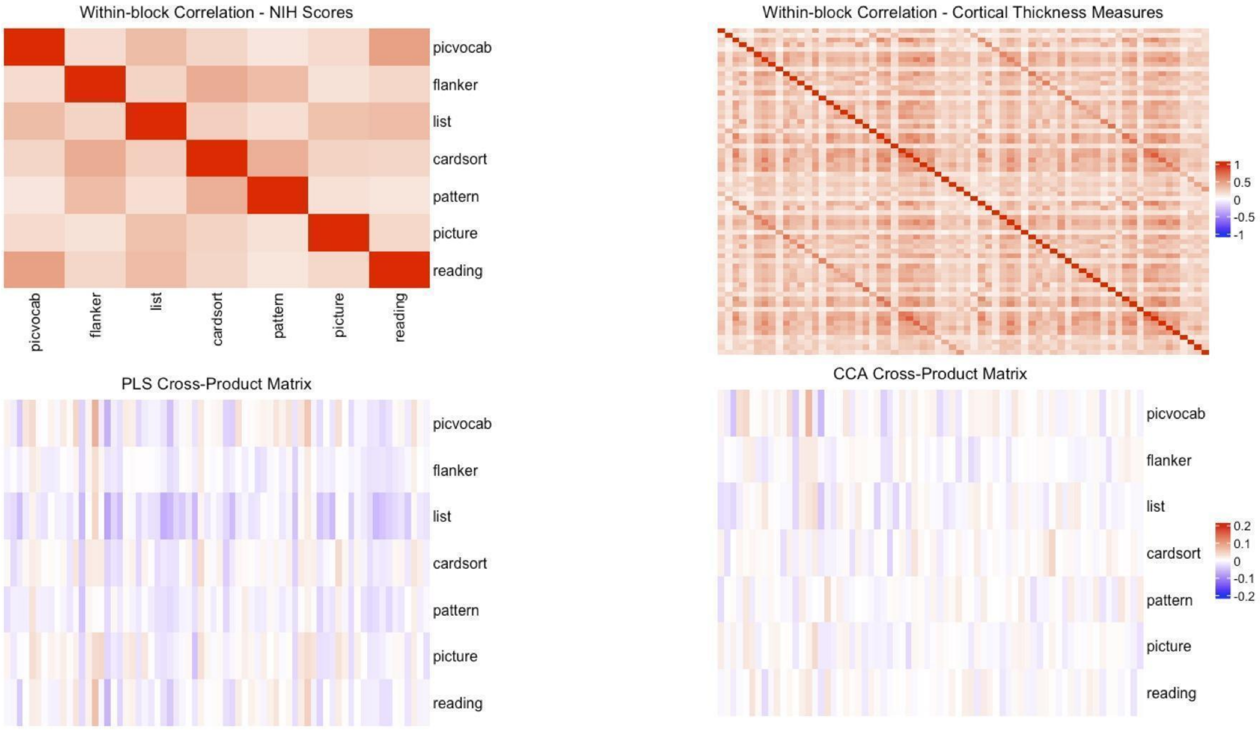

Note: The correlation figures depict zero-order Pearson correlation r-values. The X-axes labels for the cross-product matrices are the cortical thickness ROIs which were excluded from the figure for clarity of the visual representation. Similarly, the X and Y-axes labels are not included in the cortical thickness within-block correlation matrix (top right) to make the correlation matrix easier to interpret. Flanker = Flanker Task, pattern = pattern comparison processing speed task, cardsort = dimensional change card sort task, reading = oral reading recognition task, picture = picture vocabulary task, list = list sorting working memory task, picvocab = picture vocabulary task. PLS cross-product matrix = $\mathbf{R}_{\mathbf{XY}}$, CCA cross-product matrix = $\mathbf{\Omega}$.

Figure S16. Correlation plot depicting correlations between the cross-product matrices ($\boldsymbol{R}_{\boldsymbol{XY}}$ and $\boldsymbol{\Omega}$) that include total cortical volume as a regressor and that do not.

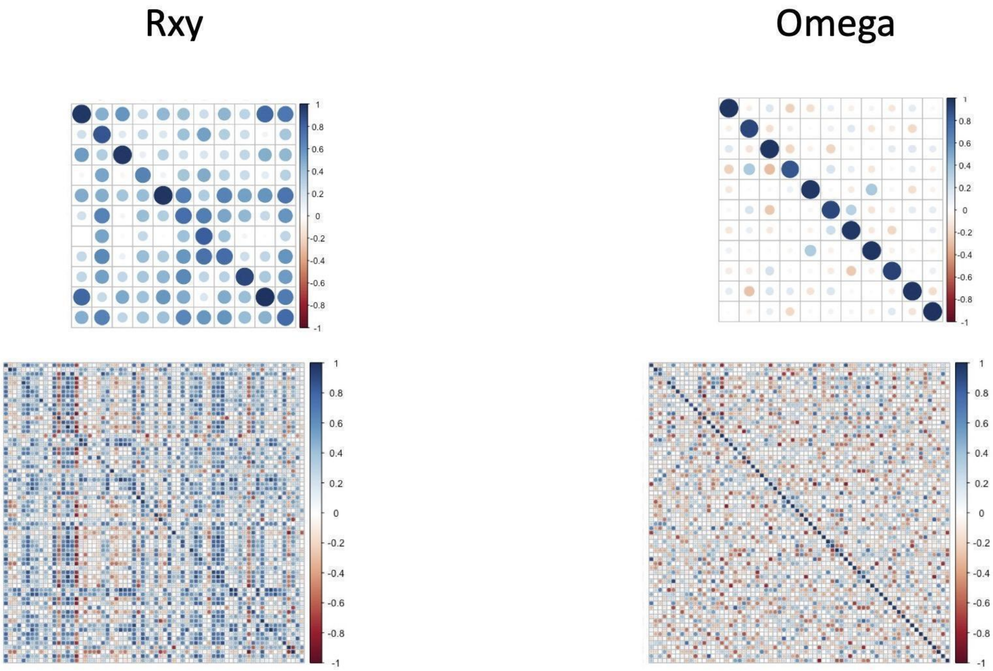

Note: The correlation figures depict zero-order Pearson correlation r-values. The diagonal of the $\mathbf{R_{XY}}$ and $\mathbf{\Omega}$ matrices across the different brain and behavioural measures are all $r > .8$ suggesting that the relationships identified would be similar regardless of regressing out total brain volume or not.

Figure S17. Distributions of residuals of the normalized CBCL and NIH scores

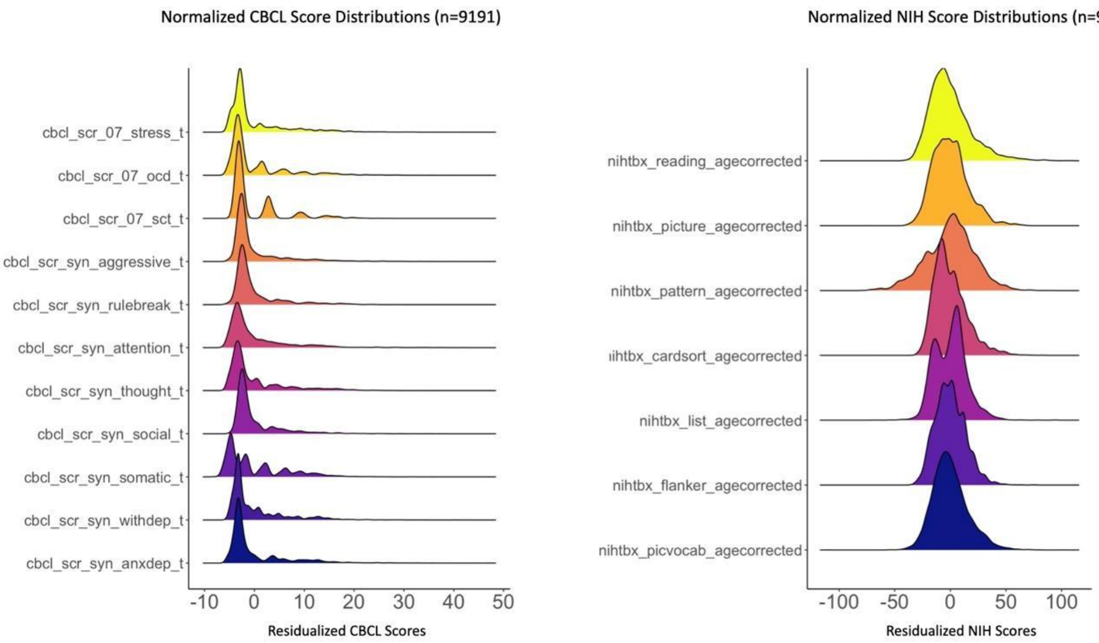

Figure S18. Comparison of the within- and between-block matrices when implementing the normalized CBCL matrix versus raw CBCL matrix

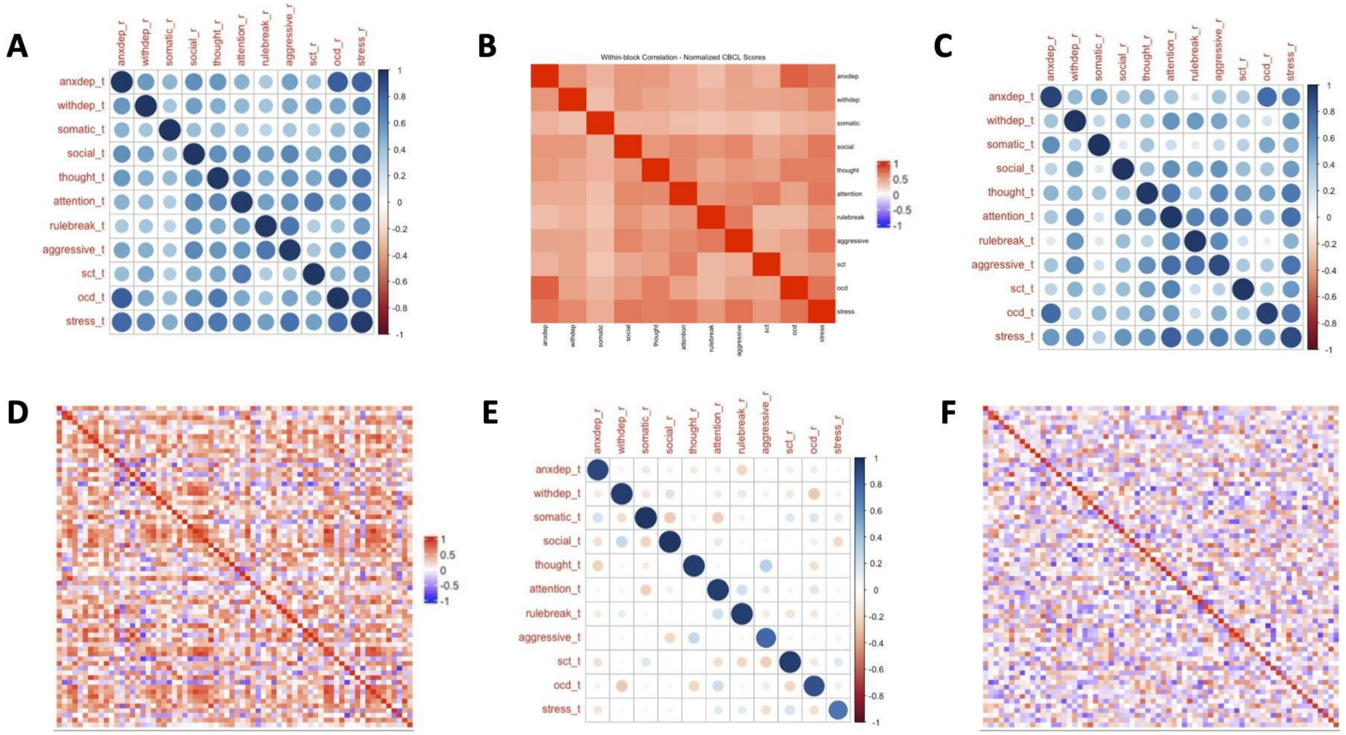

Note: This set of figures depicts the correlations between the various matrices required in CCA and PLS analyses when using the normalized CBCL scores and raw CBCL scores. Figure A depicts the correlations between the normalized (t-scores; rows) and raw CBCL scores (columns). As can be seen, the diagonal of the matrix shows very strong correlations (r>0.9) indicating strong correlations between the normalized and raw scores, as expected. Figure B depicts the within-block correlation matrix of the normalized CBCL scores. These correlations are qualitatively similar to those found in the main CBCL-brain analysis shown in Figure S12. Figure C shows the correlation of the behavioural variables from the R_{XY} cross-product matrix when using either the normalized CBCL scores (t-scores; rows) or raw CBCL scores (columns). Figure D shows the correlation of the cortical thickness variables from the R_{XY} cross-product matrix when using either the normalized CBCL scores (t-scores; rows) or raw CBCL scores (columns). Figure E shows the correlation of the behavioural variables from the Ω cross-product matrix when using either the normalized CBCL scores (t-scores; rows) or raw CBCL scores (columns). Figure F shows the correlation of the cortical thickness variables from the Ω cross-product matrix when using either the normalized CBCL scores (t-scores; rows) or raw CBCL scores (columns).

Figure S19. Comparison of the within- and between-block matrices when implementing the age-adjusted NIH matrix versus raw/unadjusted NIH matrix

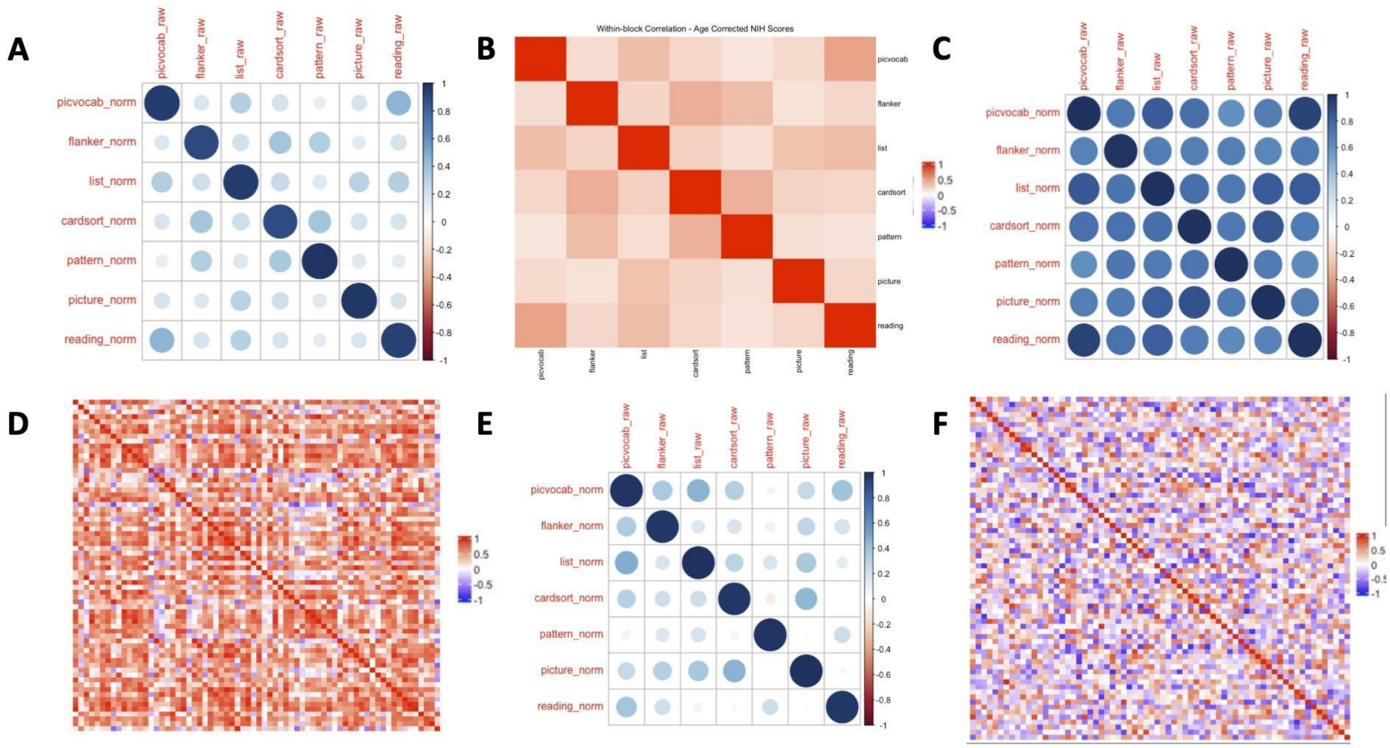

Note: This set of figures depicts the correlations between the various matrices required in CCA and PLS analyses when using the age-adjusted NIH scores and raw NIH scores. Figure A depicts the correlations between the age-adjusted (rows) and raw NIH scores (columns). As can be seen, the diagonal of the matrix shows very strong correlations (r>0.9) indicating strong correlations between the age-adjusted and raw scores, as expected. Figure B depicts the within-block correlation matrix of the age-adjusted NIH scores. These correlations are qualitatively similar to those found in the main NIH-brain analysis shown in Figure S15. Figure C shows the correlation of the behavioural variables from the R_{XY} cross-product matrix when using either the age-adjusted scores (rows) or raw NIH scores (columns). Figure D shows the correlation of the cortical thickness variables from the R_{XY} cross-product matrix when using either the age-adjusted NIH scores (rows) or raw NIH scores (columns). Figure E shows the correlation of the behavioural variables from the Ω cross-product matrix when using either the age-adjusted NIH scores (t-scores; rows) or raw NIH scores (columns). Figure F shows the correlation of the cortical thickness variables from the Ω cross-product matrix when using either the age-adjusted NIH scores (t-scores; rows) or raw NIH scores (columns).

Figure S20. Reproducibility of loadings from the CBCLnorm-brain and NIHnorm-brain analyses

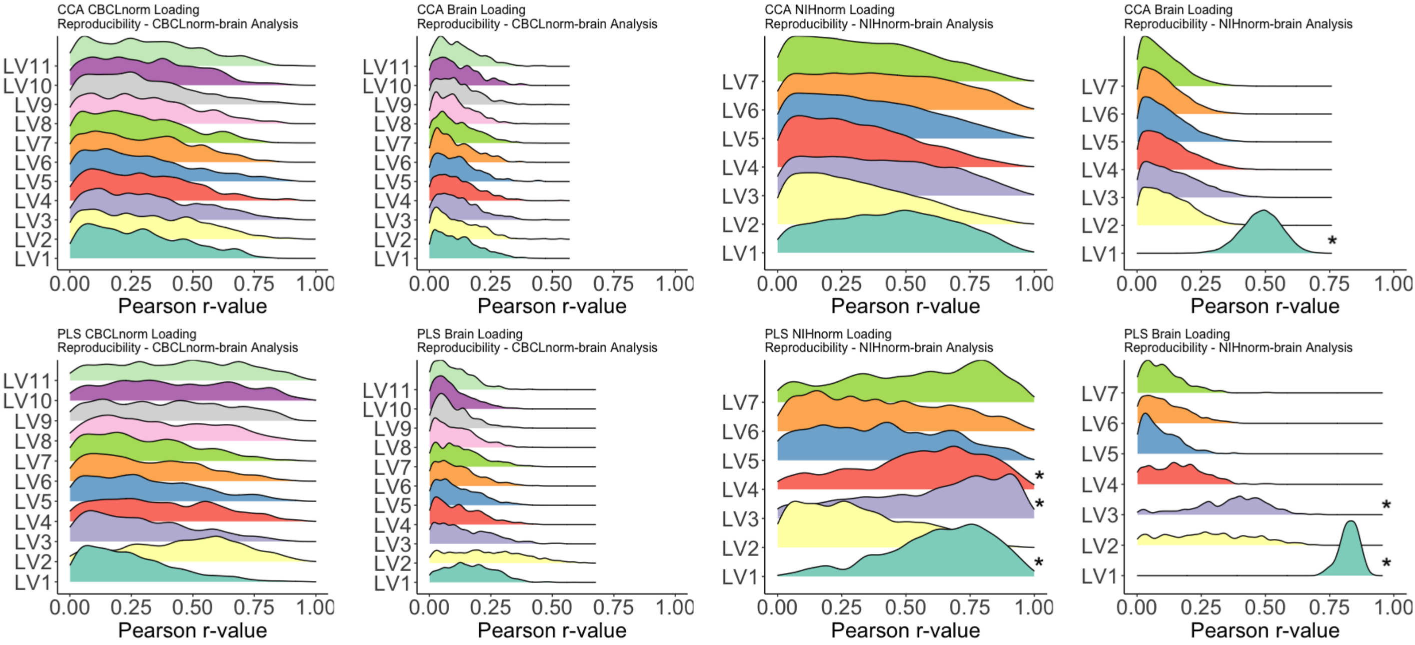

Note: This figure depicts the distributions of the resampled loadings from the split-half analysis for the CBCLnorm-brain and NIHnorm-brain analyses. The x-axis from the split-half distributions are the Pearson correlation coefficients between respective loadings from each split-half analysis (e.g., U_1 and U_2 from the analysis comparing X_1 and Y_1 and separately, X_2 and Y_2). In the CBCLnorm-brain analysis, the distribution of Pearson correlation coefficients varied between 0-0.5 with a large proportion centering around 0 for the majority of LVs, indicating minimal correspondence between respective loadings from the split-halves. This suggests that characteristics of participants are highly influential in the loadings derived from CCA or PLS models in the CBCLnorm-brain analysis, similar to that of the main CBCL-brain analysis. In the NIHnorm-brain analysis, the distribution of Pearson correlation coefficients for most of the LV_1 loadings are centered around r=0.5-0.8, indicating high correspondence between respective split-halves. Asterisks indicate the LVs which showed a distribution with a Z-score greater than 1.96.

Figure S21. Distributions of residuals of the clinical (BIS/BAS, UPPS-P) and cognitive (RAVLT) scores used in the post-hoc analysis

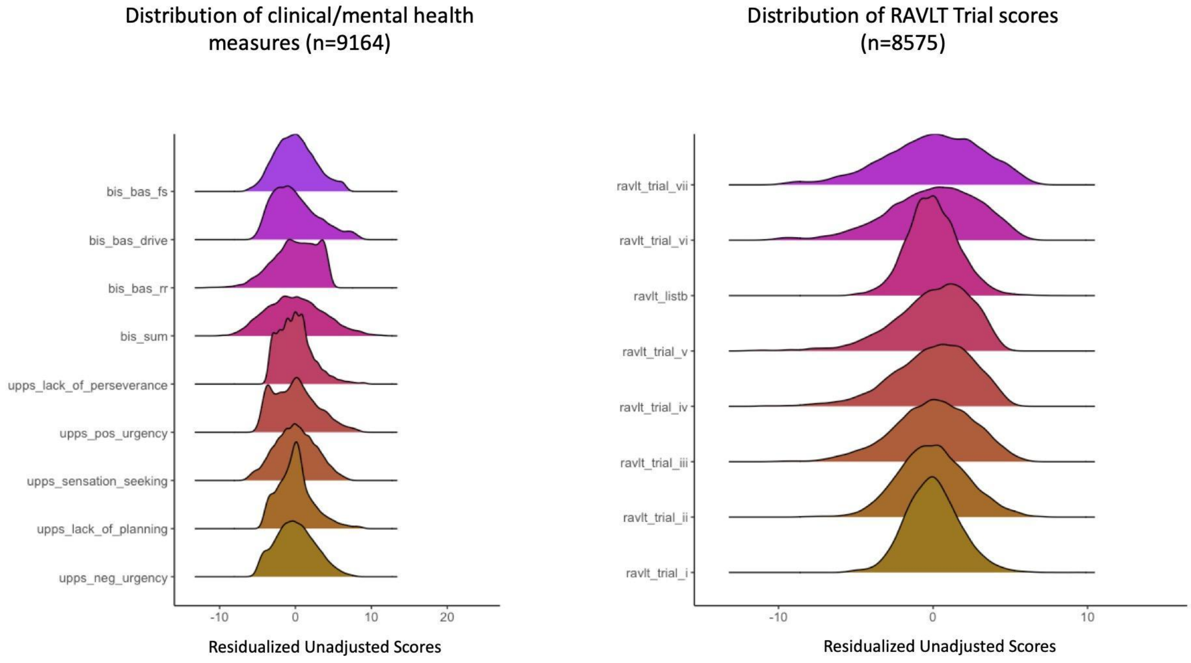

Figure S22. Correlation plots depicting the Pearson correlations between the clinical/mental health measures (CBCL, BIS/BAS, UPPS-P) and cognitive measures (NIH Toolbox, RAVLT)

Correlation plot between all clinical/mental health measures

Correlation plot between all neurocognitive measures

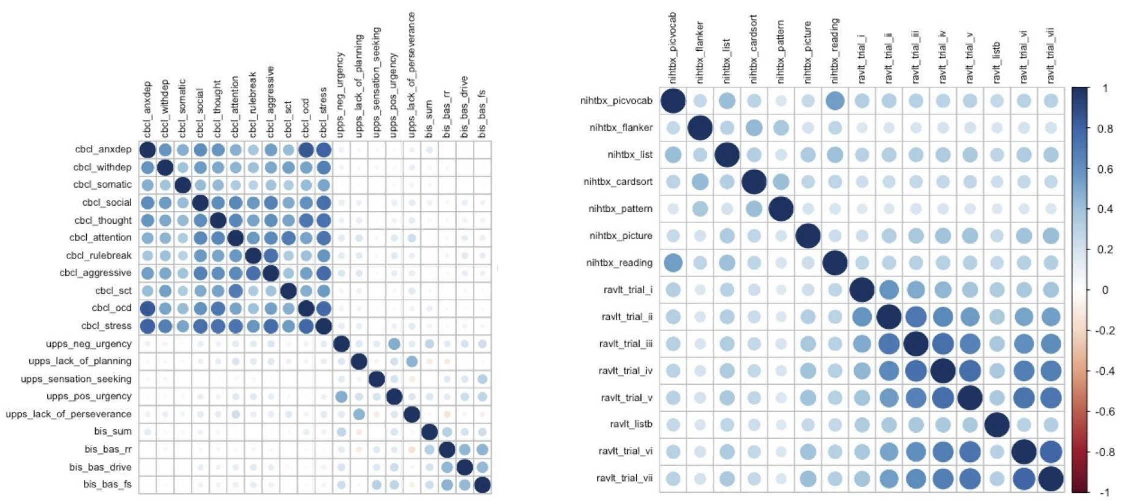

Note: OCD = obsessive compulsive symptoms, withdep = withdrawn/depression symptoms, sct = sluggish-cognitive-tempo, anxdep = anxious/depressive symptoms, rulebreak = rule breaking behaviour, rr=reward responsiveness, fs=fun seeking.

Figure S23. Cross-product matrices (R_{XY} and Ω) between the clinical measures (BIS/BAS, UPPS-P) and cortical thickness, and between the cognitive measures (RAVLT Trial Scores) and cortical thickness.

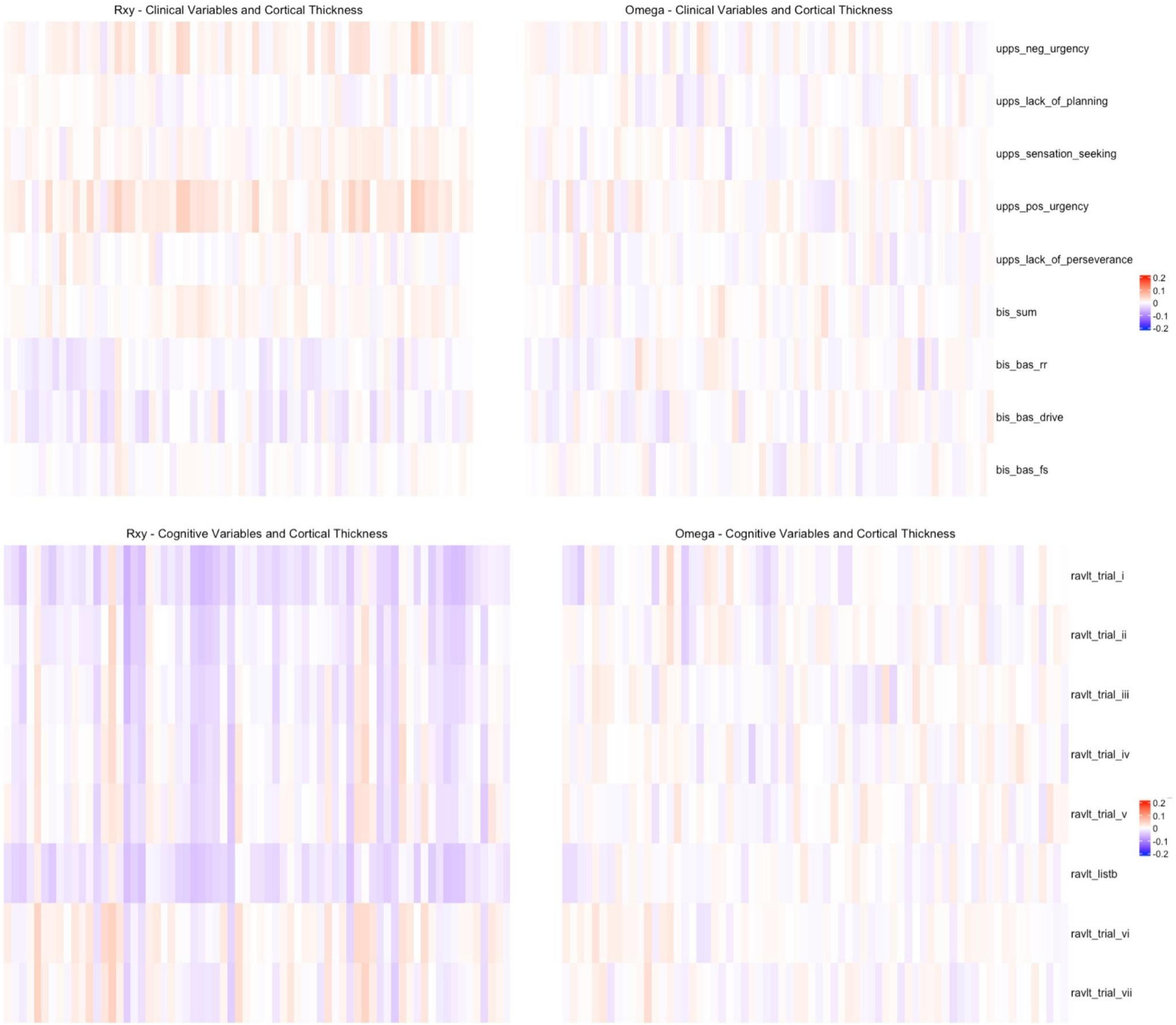

Note: neg_urgency=negative urgency, pos_urgency=positive urgency, rr=reward responsiveness, fs=fun seeking.

Figure S24. Reproducibility of the loadings from the clinical-brain and cognitive-brain analyses

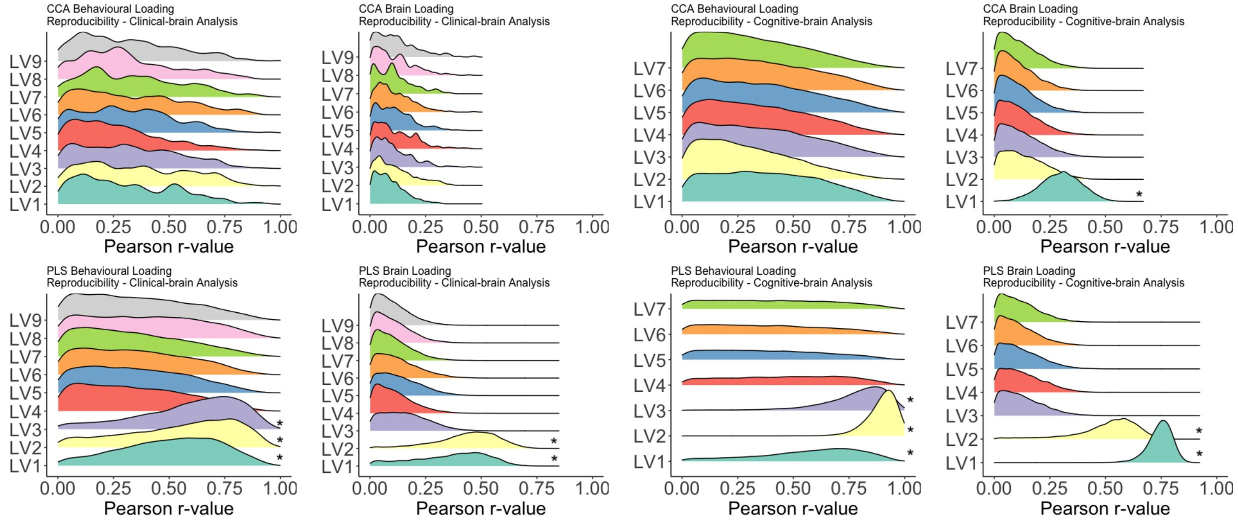

Note: This figure depicts the distributions of the resampled loadings from the split-half analysis for the clinical-brain and cognitive-brain analyses. The x-axis from the split-half distributions are the Pearson correlation coefficients between respective loadings from each split-half analysis (e.g., U_1 and U_2 from the analysis comparing X_1 and Y_1 and separately, X_2 and Y_2). In the clinical-brain analysis, the PLS-derived models showed evidence of reproducibility for the brain and behavioural loadings. However, the CCA-derived models do not show evidence of reproducibility. In the cognitive-brain analysis, PLS-derived models produced reproducible brain and behavioural loadings for LV_1 whereas, CCA-derived models produced reproducible brain, but not behavioural loadings. The PLS-derived loadings were also found to be reproducible for $LV_{2,3}$. Asterisks indicate the LVs which showed a distribution with a Z-score greater than 1.96.

6. Tables

Table S1. Demographic Characteristics of the ABCD main sample analyzed and the SES and head injury subsamples

	1. Included ABCD Sample (n=9191)		2. SES Sample (n=8399)		3. No Head Injury Sample (n=8139)		F-score, p-value
	Mean [range]	SD	Mean [range]	SD	Mean [range]	SD	
Age (in months)	118.9 [107-133]	7.4	118.9 [107-133]	7.4	118.9 [107-133]	7.4	F=0.041, p=0.96
CBCL Total Score (raw)	18.56 [0-139]	18.22	18.49 [0-139]	18.16	17.7 [0-139]	17.4	F=5.38, p=0.005, 1,2>3
CBCL Internalizing Score (raw)	5.18 [0-51]	5.62	5.18 [0-51]	5.58	4.98 [0-51]	5.42	F=3.47, p=0.03, 1,2>3
CBCL Externalizing Score (raw)	4.51 [0-49]	5.88	4.46 [0-49]	5.83	4.28 [0-49]	5.63	F=3.88, p=0.02, 1,2>3
	Total	%	Total	%	Total	%	X2, p-value
Sex (Female)	4369	47.5	4001	47.6	3974	48.8	X2=3.46, p=0.17
Household Income							X2=0.72, p=0.94
<$50K	2531	27.5	2531	27.5	2274	27.9	
$50-$100K	2381	25.9	2381	25.9	2080	25.5	
>$100K	3487	37.9	3487	37.9	3065	37.6	
Participant race/ethnicity							X2=18.9, p=0.01
White	4704	51.2	4480	53.3	4125	50.7	
Black	1360	14.8	1149	13.6	1242	15.2	
Asian	205	2.2	176	2.1	185	2.27	
Hispanic	1973	21.5	1715	20.4	1764	21.7	
Other	948	10.3	870	10.3	815	10	
Parent Education							X2=26.2, p<0.001
<HS Diploma	468	5.1	352	4.2	436	5.35	
HS Diploma/GED	887	9.65	732	8.71	810	9.95	
Some College	2389	25.9	2146	25.5	2082	25.5	
Bachelors	2287	24.9	2162	25.7	2027	24.9	
Post-Graduate	3150	34.3	3003	35.7	2775	34.1	

Table S2. Demographic Characteristics of Participants Excluded from the current study

	Included ABCD Sample (n=9191)		Excluded ABCD Sample (n=2613)		t-score, p-value
	**Mean [range]**	**SD**	**Mean**	**SD**	
Age (in months)	118.9 [107-133]	7.4	119.2 [107-132]	7.7	t=1.83, p=0.07
CBCL Total Score (raw)	18.56 [0-139]	18.22	16.8 [0-113]	16.93	t=-4.62, p<0.001
CBCL Internalizing Score (raw)	5.18 [0-51]	5.62	4.57 [0-39]	5.17	t=-5.24, p<0.001
CBCL Externalizing Score (raw)	4.51 [0-49]	5.88	4.23 [0-44]	5.75	t=-2.24, p=0.02
	**Total**	**%**	**Total**	**%**	**X2, p-value**
Sex (Female)	4369	47.5	1268	48.5	X2=0.79, p=0.37
Household Income					X2=6.29, p=0.04
<$50K	2531	27.5	667	27.8	
$50-$100K	2381	25.9	676	28.1	
>$100K	3487	37.9	1058	44.1	
Participant race/ethnicity					X2=37.4, p<0.00[illegible]
White	4704	51.2	1445	55.5	
Black	1360	14.8	393	15.1	
Asian	205	2.2	46	1.8	
Hispanic	1973	21.5	426	16.4	
Other	948	10.3	295	11.3	
Parent Education					X2=8.87, p=0.06
<HS Diploma	468	5.1	114	4.4	
HS Diploma/GED	887	9.65	232	8.9	
Some College	2389	25.9	667	25.6	
Bachelors	2287	24.9	715	27.4	
Post-Graduate	3150	34.3	877	33.7	

Note: The acquired sample had 11,804 participants with available CBCL data. As a result, the participants considered in the "excluded ABCD Sample" (n=2613) consisted of those who had CBCL data but did not meet the remainder of the inclusion criteria. Within the subset of participants excluded from the current study, there were 2401 participants with available household income data and 2605 participants with available race/ethnicity and parent education. The percentage per category is calculated accordingly (e.g., 1445/2605 = 0.554).

Table S3. Singular Values and Variance Accounted for in each LV in the CBCL-brain analysis

Main Analysis (n=9191)

PLS		CCA	
**Singular Values**	**Variance Explained**	**Singular Values**	**Variance Explained**
0.389	81.6%	0.131	19.32%
0.107	6.13%	0.112	14.17%
0.08	3.49%	0.106	12.68%
0.066	2.36%	0.098	10.79%
0.06	1.95%	0.09	9.15%
0.049	1.34%	0.084	7.99%
0.047	1.17%	0.076	6.59%
0.036	0.07%	0.073	6.07%
0.032	0.05%	0.067	5.06%
0.025	0.04%	0.064	4.66%
0.022	0.02%	0.056	3.48%

Table S4. Singular Values and Variance Accounted for in each LV in the NIH-brain analysis

NIH Analysis (n=9034)

PLS		CCA	
**Singular Values**	**Variance Explained**	**Singular Values**	**Variance Explained**
0.429	75.50%	0.205	41.60%
0.167	11.40%	0.125	15.50%
0.124	6.32%	0.115	13.20%
0.084	2.88%	0.102	10.50%
0.064	1.67%	0.091	8.24%
0.059	1.45%	0.079	6.15%
0.042	0.07%	0.069	4.71%

Table S5. Singular Values and Variance Accounted for in each LV in the Elevated-CBCL Sample

High Psychopathology (n=1016)

**PLS**		**CCA**	
**Singular Values**	**Variance Explained**	**Singular Values**	**Variance Explained**
0.671	53.9%	0.334	14.7%
0.314	11.8%	0.315	13.1%
0.288	9.96%	0.294	11.3%
0.258	7.98%	0.287	10.8%
0.197	4.62%	0.276	10%
0.174	3.62%	0.261	8.98%
0.145	2.51%	0.246	7.94%
0.127	1.92%	0.239	7.49%
0.117	1.65%	0.216	6.11%
0.1	1.19%	0.198	5.17%
0.08	0.07%	0.182	4.34%

Table S6. Singular Values and Variance Accounted for in each LV in the Symptom Endorsement Sample

**Symptom Endorsement (n=5196)**			
**PLS**		**CCA**	
**Singular Values**	**Variance Explained**	**Singular Values**	**Variance Explained**
0.38	59%	0.18	21%
0.22	18%	0.14	13%
0.13	6.90%	0.14	12%
0.11	4.70%	0.13	10%
0.09	3.10%	0.12	9.30%
0.07	2.10%	0.11	7.80%
0.06	1.80%	0.1	6.60%
0.05	1.20%	0.1	6.20%
0.05	0.09%	0.09	5.20%
0.04	0.06%	0.08	4.40%
0.03	0.04%	0.08	3.80%

Table S7. Model fit of selected brain and behaviour variables when including age and age-squared as regressors.

Behaviour/Brain Example Variables

**CBCL Variables**	**Regressor**	**F-value**	**t-value**	**p-value**
anxiety/depression subscale	age	0.85	0.92	0.35
	age-squared	0.61	0.61	0.54
attention subscale	age	2.32	1.5	0.12
	age-squared	1.49	0.8	0.23
social problems subscale	age	5.68	2.38	0.02
	age-squared	2.91	0.38	0.054
rule-breaking subscale	age	1.47	1.21	0.22
	age-squared	1.02	0.75	0.36
**Cortical Thickness Variables**				
left lateral OFC	age	84.67	9.2	<0.001
	age-squared	42.48	0.55	0.58
left pars orbitalis	age	50.75	7.12	<0.001

	age-squared	25.25	0.57	0.56
right lingual gyrus	age	86.86	9.32	<0.001
	age-squared	44.49	1.45	0.14
right temporal pole	age	0.5	0.71	0.47
	age-squared	0.44	0.61	0.54

References

Ameis, S.H., Ducharme, S., Albaugh, M.D., Hudziak, J.J., Botteron, K.N., Lepage, C., Zhao, L., Khundrakpam, B., Collins, D.L., Lerch, J.P., Wheeler, A., Schachar, R., Evans, A.C., Karama, S., 2014. Cortical thickness, cortico-amygdalar networks, and externalizing behaviors in healthy children. Biol Psychiatry 75, 65–72. https://doi.org/10.1016/j.biopsych.2013.06.008

Dienes, K.A., Chang, K.D., Blasey, C.M., Adleman, N.E. and Steiner, H., 2002. Characterization of children of bipolar parents by parent report CBCL. Journal of Psychiatric Research, 36(5), pp.337-345.

Fino, E., Melogno, S., Iliceto, P., D'Aliesio, S., Pinto, M. A., Candilera, G., & Sabatello, U. (2014). Executive functions, impulsivity, and inhibitory control in adolescents: A structural equation model. *Advances in cognitive psychology, 10(2), 32.*

Gross, D., Fogg, L., Young, M., Ridge, A., Cowell, J. M., Richardson, R., & Sivan, A. (2006). The equivalence of the Child Behavior Checklist/1 1/2-5 across parent race/ethnicity, income level, and language. Psychological Assessment, 18(3), 313–323. https://doi.org/10.1037/1040-3590.18.3.313

Hall, P.A., Best, J.R., Beaton, E.A., Sakib, M.N. and Danckert, J., 2021. Morphology of the prefrontal cortex predicts body composition in early adolescence: cognitive mediators and environmental moderators in the ABCD Study. Social Cognitive and Affective Neuroscience

Hill, W.D., Hagenaars, S.P., Marioni, R.E., Harris, S.E., Liewald, D.C.M., Davies, G., Okbay, A., McIntosh, A.M., Gale, C.R., Deary, I.J., 2016. Molecular Genetic Contributions to Social Deprivation and Household Income in UK Biobank. Current Biology 26, 3083–3089. https://doi.org/10.1016/j.cub.2016.09.035

Jackson, D. A. (1993). Stopping rules in principal components analysis: a comparison of heuristical and statistical approaches. *Ecology*, *74*(8), 2204-2214.

Lawson, G.M., Duda, J.T., Avants, B.B., Wu, J., Farah, M.J., 2013. Associations between children's socioeconomic status and prefrontal cortical thickness. Dev Sci 16, 641–652. https://doi.org/10.1111/desc.12096

Lynam, D.R., Smith, G.T., Cyders, M.A., Fischer, S., Whiteside, S.A. (2007). The UPPS-P: a multidimensional measure of risk for impulsive behavior. *Unpublished technical report*

McIntosh, A.R., Lobaugh, N.J., 2004. Partial least squares analysis of neuroimaging data: Applications and advances. Neuroimage 23, 250–263. https://doi.org/10.1016/j.neuroimage.2004.07.020

Modabbernia, A., Janiri, D., Doucet, G.E., Reichenberg, A., Frangou, S., 2021. Multivariate Patterns of Brain-Behavior-Environment Associations in the Adolescent Brain and Cognitive Development Study. Biol Psychiatry 89, 510–520. https://doi.org/10.1016/j.biopsych.2020.08.014

Myers, L. and Sirois, M.J., 2006. Spearman correlation coefficients, differences between. Encyclopedia of statistical sciences, 12. https://doi.org/10.1002/0471667196.ess5050.pub2

Nigg, J. T. (2017). Annual Research Review: On the relations among self-regulation, self-control, executive functioning, effortful control, cognitive control, impulsivity, risk-taking, and inhibition for developmental psychopathology. *Journal of child psychology and psychiatry*, *58*(4), 361-383.

Owens, M.M., Potter, A., Hyatt, C.S., Albaugh, M., Thompson, W.K., Jernigan, T., Yuan, D., Hahn, S., Allgaier, N. and Garavan, H., 2021. Recalibrating expectations about effect size: A multi-method survey

of effect sizes in the ABCD study. PloS one 16, p.e0257535. https://doi.org/10.1371/journal.pone.0257535

Pagliaccio, D., Luking, K. R., Anokhin, A. P., Gotlib, I. H., Hayden, E. P., Olino, T. M., ... & Barch, D. M. (2016). Revising the BIS/BAS Scale to study development: Measurement invariance and normative effects of age and sex from childhood through adulthood. *Psychological assessment*, *28*(4), 429.

Piccolo, L.R., Merz, E.C., He, X., Sowell, E.R., Noble, K.G., 2016. Age-related differences in cortical thickness vary by socioeconomic status. PLoS One 11, 1–18. https://doi.org/10.1371/journal.pone.0162511

Rakesh, D., Zalesky, A., Whittle, S., 2021. Similar but distinct – Effects of different socioeconomic indicators on resting state functional connectivity: Findings from the Adolescent Brain Cognitive Development (ABCD) Study®. Dev Cogn Neurosci 51, 101005. https://doi.org/10.1016/j.dcn.2021.101005

Tollenaar, M.S., Beijers, R., Garg, E., Nguyen, T.T., Lin, D.T., MacIsaac, J.L., Shalev, I., Kobor, M.S., Meaney, M.J., O'Donnell, K.J. and de Weerth, C., 2021. Internalizing symptoms associate with the pace of epigenetic aging in childhood. Biological Psychology, 159, p.108021.

Strauss, E., Sherman, E. M., & Spreen, O. (2006). *A compendium of neuropsychological tests: Administration, norms, and commentary*. American chemical society.

Watts, A. L., Smith, G. T., Barch, D. M., & Sher, K. J. (2020). Factor structure, measurement and structural invariance, and external validity of an abbreviated youth version of the UPPS-P Impulsive Behavior Scale. *Psychological assessment*, *32*(4), 336.

Wilde, E.A., Merkley, T.L., Bigler, E.D., Max, J.E., Schmidt, A.T., Ayoub, K.W., McCauley, S.R., Hunter, J. V., Hanten, G., Li, X., Chu, Z.D., Levin, H.S., 2012. Longitudinal changes in cortical thickness in children after traumatic brain injury and their relation to behavioral regulation and emotional control. International Journal of Developmental Neuroscience 30, 267–276. https://doi.org/10.1016/j.ijdevneu.2012.01.003

Zhu, X., Ward, J., Cullen, B. et al. Phenotypic and genetic associations between anhedonia and brain structure in UK Biobank. Transl Psychiatry 11, 395 (2021). https://doi.org/10.1038/s41398-021-01522-4
